# A new distance between rankings

**DOI:** 10.1016/j.heliyon.2024.e28265

**Published:** 2024-03-20

**Authors:** Jean Dezert, Andrii Shekhovtsov, Wojciech Sałabun

**Affiliations:** a Department of Information Processing and Systems, The French Aerospace Lab - ONERA, 91120 Palaiseau, France; b National Telecommunications Institute, ul. Szachowa 1, Warsaw, 04-894, Poland

**Keywords:** Ranking, Distance, F-distance, Spearman's distance, Kemeny's distance, Frobenius' distance

## Abstract

This paper analyzes the behavior of the well-known Spearman's footrule distance (*F*-distance) to measure the distance between two rankings over the same set of objects. We show that *F*-distance is not invariant to labeling, and therefore, it suffers from a serious drawback for its use in applications. To circumvent this problem, we propose a new distance between rankings which is invariant under indexing (i.e., labeling) and appears as a good alternative to the direct use of *F*-distance between rankings, and also the invariant-under-indexing Kemeny's distance as well. We also show how our new distance can work with importance weights. Some simple examples are given to show the interest of our method with respect to the classical one based on *F*-distance and Kemeny's distance.

## Highlights

• New robust alternative for the Spearman's footrule distance.
• Based on Preference-Score Matrix and Frobenius norm.
• Satisfies invariance under indexing principle and Kemeny's axioms.

## 1 Introduction

In many multi-criteria decision-making (MCDA) problems, it is required to compare several methods to obtain a more reliable solution [26], [31]. The problem of comparison of different methods is connected with the problem of measuring the distance or the correlation between rankings obtained using different methods [19], [33].

There are many different correlation coefficients proposed to measure the difference between the two rankings. Such coefficients as Kendall Tau [18], [29], Spearman's rank correlation coefficient [32], and weighted coefficients such as Weighted Spearman's correlation [6] and Weighted Similarity rank coefficient [28] are often used in the literature to compare the rankings [31], [34]. However, those coefficients do not follow the properties of the distance definition: symmetry, separation, and triangular inequality.

In most cases, it is possible to use distance functions, such as Spearman's footrule [2], [5], Kemeny's distance [13] or generalized Minkowski distance. However, they are not fulfilling invariance under the indexing principle (IUIP). It means that a calculation of the distance between two rankings could result in different values if a set of labels is changed. This behavior is not desired and not appropriate in most cases [16].

In some works, rankings are presented as an index of the labels set or as ordered labels set [8], [25]. Researchers use this method to represent the rankings because it could be considered the most natural [10], [21]. However, in this case, calculating the distance between rankings will result in violating invariance under the indexing principle. It is also possible to represent ranking by position [4], [3]. Calculating the distance between such rankings will not violate IUIP, but creating ranking by position could be considered unnatural for some people. Therefore, we want to propose a new method for computation of the distances between ranking written by indexes, which will follow the properties of a true metric and satisfy IUIP, as well as axioms presented in [17].

The need for such distance metric is underlined with recent works, such as a proposition of $WS_{dra}$ metric by [27], which also fulfills the properties of a true metric and utilizes ranking by index to satisfy invariance under indexing principle. However, this metric utilizes an entirely different approach and can not be applied to long ranking vectors. The usage of a new metric is not limited only to measurements between different rankings. Such distance can also power distance-based methods such as TOPSIS [24] to potentially improve the decision-making quality. Additionally, it can be used in other domains besides MCDA, for example, in machine-learning-related algorithms that utilize distance functions, such as clustering [11], [13], fuzzy clustering [12] or classification [30], where newly developed distances can greatly improve existing algorithms.

The main contribution of this paper is to propose a new effective method to calculate the distance between rankings, which will be invariant to the labeling of decision alternatives. The proposed approach fulfills IUIP and could be potentially extended and generalized to other distances. Additionally, it follows the properties of a true metric, i.e., symmetry, separation, and triangular inequality. We also prove that our proposed approach satisfies Kemeny's axioms for rankings distance metric [16]. The useful and desired properties of the proposed distance will ensure its applicability not only in the MCDA domain but also for other distance-related problems, such as distance-based machine-learning algorithms.

The rest of the paper is structured as follows. In Section 2, all necessary definitions and notations are provided. In Section 3, we describe the footrule distance proposed by Spearman and describe the problem of labeling invariance. Section 4 describes the invariance under the indexing principle and how it is violated when Spearman's *F*-distance is used. Next, in Section 5, we propose a new distance that overcomes the IUIP problem, and we compare it to Kemeny's distance. We all discuss Kemeny's axiomatic and extend our new distance for also working with importance weights. Finally, in Section 6, we draw conclusions and propose future work directions.

## 2 Definitions and notations

### 2.1 Distance

Let *X* be a set. A function $d:X\times X\to R_{+}$ is called a distance (or dissimilarity, or metric) on *X*, if the properties (1) - (3) hold [7] (Chap. 1)

1. Symmetry:
  (1) ∀x,y∈X,d(x,y)=d(y,x),
2. Separation (identity of indiscernibles):
  (2) ∀x,y∈X,d(x,y)=0⇔x=y,
3. Triangular inequality:
  (3) ∀x,y,z∈X,d(x,z)≤d(x,y)+d(y,z).

A set *X* endowed with a distance is named a metric space, or a distance space.

There exist many distances proposed in the literature, see [7] for a good survey, but the most common ones are just specific cases of the Minkowski distance [20] of order *p* (where p≥1 is an integer) between two points,^1^ $x≜[x_{1},x_{2},\ldots ,x_{n}]$ and $y≜[y_{1},y_{2},\ldots ,y_{n}]$ of the space $R^{n}$ which is defined by (4)

(4) $$d_{p}(x,y)=||x-y|{|}_{p}≜{\left[\sum_{i=1}^{n}|x_{i}-y_{i}{|}^{p}\right]}^{1/p}.$$

In practice, the Minkowski distance is used with p=1 or p=2. For p=1 the Minkowski distance is known as the Manhattan distance, or the city-clock distance, which is equal to (5)

(5) $$d_{1}(x,y)=||x-y|{|}_{1}=\sum_{i=1}^{n}|x_{i}-y_{i}|.$$

For p=2 the Minkowski distance is called the Euclidean distance given by (6)

(6) $$d_{2}(x,y)=||x-y|{|}_{2}=\sqrt{\sum_{i=1}^{n}{\left(x_{i}-y_{i}\right)}^{2}}.$$

In the limiting case of *p* reaching infinity, we obtain the Chebyshev distance (7)

(7) $$d_{\infty }(x,y)=||x-y|{|}_{\infty }=\max_{i=1,\ldots ,n}|x_{i}-y_{i}|.$$

### 2.2 Preference order

Let's consider two objects denoted by $x_{i}$ and $x_{j}$. If the object $x_{i}$ is more preferred than object $x_{j}$, we denote this preference as $x_{i}≻x_{j}$. If the object $x_{i}$ is less preferred than object $x_{j}$, we denote this preference as $x_{i}≺x_{j}$. If objects $x_{i}$ and $x_{j}$ have no preference order, that is, neither preference $x_{i}≻x_{j}$ or $x_{j}≻x_{i}$ is valid, then we write $x_{i}=x_{j}$ for characterizing the indifference (or ex aequo preferences, or tie) in the choice between $x_{i}$ and $x_{j}$.

### 2.3 Reference set

Let $X=\{x_{1},\ldots ,x_{n}\}$ be a set^2^ of items (or elements, or objects) to be ranked by an expert (either by a human expert opinion or by an artificial expert system). The set *X* is called the reference set of objects if each object is labeled with a distinct given integer index i=1,…,n. Because there are many ways to commit indexes to objects, the reference set is not unique. For instance, if we consider four objects *A*, *B*, *C* and *D* the reference set can be chosen either as $X=\{x_{1}=A,x_{2}=B,x_{3}=C,x_{4}=D\}$, $Y=\{y_{1}=D,y_{2}=C,y_{3}=B,y_{4}=A\}$, or defined by any other choice of permutation of indexes 1, 2, 3 and 4. Note that a reference set is a non-ordered set of elements, which means that the way to list the elements of the reference set does not matter. Hence in this example, the sets with all permutations of indexes i=1,2,3,4 like $\left\{x_{1},x_{2},x_{3},x_{4}\right\}$, $\left\{x_{2},x_{1},x_{3},x_{4}\right\}$, $\left\{x_{3},x_{2},x_{1},x_{4}\right\}$, $\left\{x_{4},x_{1},x_{2},x_{3}\right\}$, etc represent all the same reference set *X*.

### 2.4 Ranking-index and ranking

A ranking-index is an ordered *n*-uple associated to a reference set *X*. We denote it $r_{X}=(r_{X}(1),\ldots ,r_{X}(n))$, where $r_{X}(i)$ is the rank (or preference order) associated with the element $x_{i}$ of *X*. $r_{X}$ is a total (i.e., strict) ranking index if there is no equality of preference between some elements of *X*, which means that there is no tie in the preferences among some objects of the reference set. The sum of values of a total ranking index of size *n* is the sum of the first *n* natural numbers, which is equal to n(n+1)/2. A ranking $R_{r_{X}}(X)$ is a permutation of objects of the set *X*, which is determined by a preference ordering specified by a ranking-index *n*-uple $r_{X}$. Therefore, a ranking $R_{r_{X}}(X)$ associated with total ranking-index $r_{X}$ a is a perfectly ordered set of objects. Each ranking of a set *X* given by an expert *s* (a source of information) is denoted by $R_{r_{X,s}}(X)$, and its ranking-index by $r_{X,s}=(r_{X,s}(1),\ldots ,r_{X,s}(n))$, where $r_{X,s}(i)$ is the rank associated with the element $x_{i}$ by the *s*-th source of information (for s=1,2,…,S). Without loss of generality and by convention, the first element of this ordered set $R_{r_{X,s}}(X)$ will be considered as the most preferred object by the *s*-th expert, the second element will be considered as the second best-preferred object by this expert, etc.

### 2.5 Example 1

Consider the reference set $X=\{x_{1},x_{2},x_{3}\}$, and suppose an expert^3^ commits to the element $x_{2}$ his best preference, to the element $x_{3}$ his second best preference, and to $x_{1}$ his last preference. This expert's preference order is denoted as $x_{2}≻x_{3}≻x_{1}$. His corresponding ranking-index is thus written as $r_{X}=(r_{X}(1),r_{X}(2),r_{X}(3))=(2,3,1)$, where $r_{X}(1)=2$ means that the 1st preferred object is $x_{2}$, $r_{X}(2)=3$ means that the 2nd preferred object is $x_{3}$, and $r_{X}(3)=1$ means that the 3rd preferred object is $x_{1}$. Because $r_{X}=(2,3,1)$, the ranking of *X* for this expert is thus the ordered set $R_{r_{X}}(X)=\{x_{2},x_{3},x_{1}\}$.

### 2.6 Ranking-index including some ties

When two (or more) objects have the same preference order (i.e., their ranks are ex aequo) we write them as a non-ordered list of their indexes between inner left and right parentheses. For instance, if we consider four objects *A*, *B*, *C* and *D* and we define the reference set as $X=\{x_{1}=A,x_{2}=B,x_{3}=C,x_{4}=D\}$, then the preference order with one tie between two objects like A≻(B=D)≻C corresponds to the tied ranking-index $r_{X}=(1,(2,4),3)$ which can also be written equivalently as $r_{X}=(1,(4,2),3)$. This notation means that object $x_{1}=A$ is the most preferred object, the objects $x_{2}=B$ and $x_{4}=D$ have ex aequo second-best preference, and $x_{3}=C$ is the least preferred object. In our interpretation and with our notation, the rank of the object $x_{1}=A$ is 1, the rank of objects $x_{2}=B$ and $x_{4}=D$ are both equal to 2 because of the tie they both appear in the second component of the (tied) ranking-index $r_{X}=(1,(4,2),3)$, and the rank of the least preferred object $x_{3}=C$ is 3. Note that the sum of rank values of all objects is then 1+2+2+3=9, whereas it would be 1+2+3+4=10 if no tie occurs. The corresponding tied ranking set for this tied preference order A≻(B=D)≻C is denoted by $R_{r_{X}}(X)=\{x_{1},(x_{2},x_{4}),x_{3}\}$, and it is worth noting that in this case $R_{r_{X}}(X)$ is only a partially ordered because of the preference tie between objects $x_{2}$ and $x_{4}$.

If we consider another type of tie in preference, say A≻(B=C=D) (one tie with three objects), we write $r_{X}=(1,(2,3,4))$. If one considers the other tied preference order (A=B)≻(C=D) (two ties with two objects) we write $r_{X}=((1,2),(3,4))$. In the whole indifference case where the (degenerate, or fully tied) preference order is A=B=C=D (one tie with all the four objects), we write $r_{X}=((1,2,3,4))$. Note that the double parentheses notation is very important in order to identify the ties (if any) in the ranking index.

Instead of using our previous interpretation and notations, some users working on ranking problems prefer to commit average rank to objects involved in a tie, for instance if we consider the reference set $X=\{x_{1}=A,x_{2}=B$, $x_{3}=C,x_{4}=D\}$ and a tied preference like A≻(B=D)≻C, they actually consider that both orders A≻B≻D≻C and A≻D≻B≻C are valid simultaneously. In this case, they consider that object $x_{1}=A$ has rank 1 being the first/most preferred object, the rank of the object $x_{2}=B$ is both 2 and 3 (so they take its middle-rank value 2.5), the rank of the object $x_{4}=D$ is also both 2 and 3 (so they take it also its middle value 2.5), and the rank of least preferred object $x_{3}=C$ is 4. With this classical method, the sum of ranks of objects is, for this example 1+2.5+2.5+4=10, which is the same as the sum of rank values 1+2+3+4=10 if no tie occurs in the preference order. This second method for dealing with ties is commonly used in practice, but the interpretation of non-integer values for ranks is difficult and questionable because it is clear that based on the sum of these rank values, we cannot discriminate if a preference order is strict/total (i.e., having no tie), or only partial (including ties) contrary to the previous method which is, we think, disputable.

## 3 Spearman $L_{1}$-distance between rankings

Spearman [32] proposed to use the $L_{1}$ distance to measure the distance between two ranking indexes. This distance is referred to as Spearman's footrule distance in [9]. It is also known as *F*-distance in the literature.

### 3.1 Definition

The *F*-distance (i.e., Spearman $L_{1}$-distance) is the sum of the absolute differences between the components of the ranking indexes (i.e., *n*-uples). Suppose we have two experts providing two ranking-index *n*-uples $r_{X,1}$ and $r_{X,2}$ defined over the same reference set of objects $X=\{x_{1},\ldots ,x_{n}\}$, then the Spearman's footrule distance between $r_{X,1}$ and $r_{X,2}$ is defined as follows (8):

(8) $$F(r_{X,1},r_{X,2})≜\sum_{i=1}^{n}|r_{X,1}(i)-r_{X,2}(i)|,$$

where $r_{X,1}$ and $r_{X,2}$ are total ranking-indexes over the reference set of objects *X*.

The *F*-distance is nothing but an $L_{1}$-distance and a metric, and it is possible to compute it in linear time.

### 3.2 Normalization of the *F*-distance

The *F*-distance can be normalized in [0,1] by dividing $F(r_{X,1},r_{X,2})$ by its maximum value $F^{\max}(n)$ which is obtained by calculating the *F*-distance between the two fully contradictory ranking-indexes $r_{X,1}=(1,2,3,\ldots ,n-1,n)$ and $r_{X,2}=(n,n-1,\ldots ,3,2,1)$.

The maximum of *F*-distance is expressed as (9)

(9) $$F^{\max}(n)=\sum_{i=1}^{n}|i-(n+1-i)|=\sum_{i=1}^{n}|2i-(n+1)|.$$

Two cases must be analyzed to calculate $F^{\max}(n)$:

• Case 1: *n* is an even number
  If *n* is an even number then n=2m, and in this case we have m=n/2 and $F^{\max}(n)$ can be decomposed as (10)
  (10) $$F^{\max}(n)=\sum_{i=1}^{m}|2i-(2m+1)|+\sum_{i=m+1}^{2m}|2i-(2m+1)|$$
  We note that the sums $S_{1}=\sum_{i=1}^{m}|2i-(2m+1)|$ and $S_{2}=\sum_{i=m+1}^{2m}|2i-(2m+1)|$ are actually equal because the terms are equal when index increases in $S_{1}$ and decreases $S_{2}$. For instance for the first term (for i=1) of $S_{1}$ we get |2−(2m+1)|=|−2m+1| and for the last term (for i=2m) of $S_{2}$ we get |2(2m)−(2m+1)|=|2m−1| and these two terms |−2m+1| and |2m−1| are equal. For the second term (for i=2) of $S_{1}$ we get |4−(2m+1)|=|3−2m| and for the penultimate term (for i=2m−1) of $S_{2}$ we get |2(2m−1)−(2m+1)|=|2m−3| and these two terms |3−2m| and |2m−3| are equal, etc. For the last term (for i=m) of $S_{1}$ we get |2m−(2m+1)|=|−1|=1 and for the first term of $S_{2}$ we get |2(m+1)−(2m+1)|=|1|=1. Therefore, if *n* is an even number, we have (11)
  (11) $$F^{\max}(n)=2\cdot \sum_{i=1}^{m}|2i-(2m+1)|=2\cdot S_{1}.$$
  Because |2i−(2m+1)| is always an odd number the sum $S_{1}=\sum_{i=1}^{m}|2i-(2m+1)|$ is equal to the sum of the *m* first odd positive numbers, and it is given by $S_{1}=m^{2}={\left(\frac{n}{2}\right)}^{2}=\frac{n^{2}}{4}$. Therefore, we finally get for n=2m (the even number case) (12)
  (12) $$F^{\max}(n)=2\cdot \frac{n^{2}}{4}=\frac{n^{2}}{2}.$$
• Case 2: *n* is an odd number
  If *n* is an odd number then n=2m+1, and $F^{\max}(n)$ can always be decomposed as (13)
  (13) $$F^{\max}(n)=\sum_{i=1}^{m}|2i-(2m+2)|+\sum_{i=m+2}^{2m+1}|2i-(2m+2)|.$$
  Similarly to the previous case when n=2m, one can also verify that the sum $S_{3}=\sum_{i=1}^{m}|2i-(2m+2)|$ and $S_{4}=\sum_{i=m+2}^{2m+1}|2i-(2m+2)|$ are equal, and therefore we have (14)
  (14) $$F^{\max}(n)=2\cdot \sum_{i=1}^{m}|2i-(2m+2)|=2\cdot S_{3}.$$
  Because |2i−(2m+2)| is always an even number the sum $S_{3}=\sum_{i=1}^{m}|2i-(2m+1)|$ is equal to the sum of the *m* first even positive numbers, and it is given by $S_{3}=m(m+1)$. Therefore, for n=2m+1 (the odd number case), we get (15)
  (15) $$F^{\max}(n)=2m(m+1).$$
  Because n=2m+1, one has $m=\frac{1}{2}(n-1)$. Replacing the expression of *m* in (15), we get (16)
  (16) $$F^{\max}(n)=\frac{1}{2}(n-1)(n+1).$$

In summary, the normalized Spearman *F*-distance between two rankings $\overset{˜}{F}(r_{X,1},r_{X,2})$ is given by (17)

(17) $$\overset{˜}{F}(r_{X,1},r_{X,2})=\frac{F(r_{X,1},r_{X,2})}{F^{\max}(n)}=\left\{ \begin{array}{c} \frac{2}{n^{2}}\sum_{i=1}^{n}|r_{X,1}(i)-r_{X,2}(i)|,\;\text{(if }n\text{ even),}\\ \frac{2}{\left(n-1)(n+1\right)}\sum_{i=1}^{n}|r_{X,1}(i)-r_{X,2}(i)|,\;\text{(if }n\text{ odd)}. \end{array}\right.$$

If the normalized *F*-distance equals one it means two totally different rankings, and if it equals zero it means identical rankings.

### 3.3 Example 2 (with Spearman's *F*-distance)

Consider n=4 different elements (or objects) denoted as *A*, *B*, *C*, and *D* (for example, cars, bikes, houses, wines, or whatever). Suppose that the reference set of objects is chosen as $X=\{x_{1}=A,x_{2}=B,x_{3}=C,x_{4}=D\}$. We consider two experts providing each the ranking-indexes $r_{X,1}=(4,2,1,3)$ and $r_{X,2}=(2,3,4,1)$ expressing their own preference choice of these objects based on some own criteria. The ranking-index $r_{X,1}=(4,2,1,3)$ means that the first expert has the preference order D≻B≻A≻C, whereas $r_{X,2}=(2,3,4,1)$ means that the second expert has the preference order B≻C≻D≻A. Therefore, the ranked (ordered) sets are respectively equal to $R_{r_{X,1}}(X)=\{x_{4},x_{2},x_{1},x_{3}\}=\{D,B,A,C\}$ and equal to $R_{r_{X,2}}(X)=\{x_{2},x_{3},x_{4},x_{1}\}=\{B,C,D,A\}$. The *F*-distance between these two ranking-indexes $r_{X,1}$ and $r_{X,2}$ is (18)

(18) $$F(r_{X,1},r_{X,2})=\sum_{i=1}^{4}|r_{X,1}(i)-r_{X,2}(i)|=|4-2|+|2-3|+|1-4|+|3-1|=2+1+3+2=8,$$

and (19)

(19) $$\overset{˜}{F}(r_{X,1},r_{X,2})=(\frac{2}{n^{2}})\cdot F(r_{X,1},r_{X,2})=\frac{2}{16}\cdot 8=1.$$

Because $\overset{˜}{F_{X}}(r_{X,1},r_{X,2})=1$ (which is the maximum normalized distance), it means that the rankings based on $r_{X,1}$ and $r_{X,2}$ are totally different and inconsistent. A priori, we may consider that this result makes sense because these two rankings, which reflect the preference orders D≻B≻A≻C and B≻C≻D≻A, are very different, and because they do not share a same object at the same rank of the preference order because the two rankings are (20) and (21).

(20) $$R_{r_{X,1}}(X)=\{D,B,A,C\},$$

(21) $$R_{r_{X,2}}(X)=\{B,C,D,A\}.$$

However, we can already suspect a problem in this *F*-distance measure because it does not capture well some partial consistencies between preferences orders ${\text{Pref}}_{1}≜D≻B≻A≻C$ and ${\text{Pref}}_{2}≜B≻C≻D≻A$ expressed by the experts. For example, in ${\text{Pref}}_{1}$ and in ${\text{Pref}}_{2}$ we have the preference B≻A satisfied, as well all the preference D≻A. So it seems counter-intuitive to consider the rankings $R_{r_{X,1}}(X)=\{D,B,A,C\}$ and $R_{r_{X,2}}(X)=\{B,C,D,A\}$ as totally different and fully inconsistent.

### 3.4 Calculation of *F*-distance when ties occur

In our previous example 2, we considered rankings with no ties, and we did calculate the *F*-distance based on formula (8) without difficulty. For applying formula (8) when ties occur in the ranking index, we must proceed differently for indexes where a tie occurs. The classical method is to calculate the average value of all indexes involved in a tie and replace the indexes of the tie with their average value (which can be a noninteger index). Then, the formula (8) is used. For instance, if we consider the reference set $X=\{x_{1}=A,x_{2}=B,x_{3}=C,x_{4}=D\}$ and the preference order $Pref_{1}=B≻A≻(D=C)$ and the second preference order $Pref_{2}=B≻(A=C)≻D$, then the corresponding ranking-indexes are respectively given by $r_{X,1}=(2,1,(3,4))$ and $r_{X,2}=(2,(1,3),4)$. Replacing the indexes appearing in ties by their average value, we now consider the modified ranking-indexes $r_{X,1}^{\prime }=(2,1,3.5,3.5)$ and $r_{X,2}^{\prime }=(2,2,2,4)$ in the *F*-distance formula and we obtain (22)

(22) $$F(r_{X,1},r_{X,2})=\sum_{i=1}^{4}|r_{X,1}^{\prime }(i)-r_{X,2}^{\prime }(i)|=|2-2|+|1-2|+|3.5-2|+|3.5-4|=0+1+1.5+0.5=3.$$

This method for dealing with ties in ranking indexes is actually disputable because the interpretation and justification of noninteger indexes are unclear, and the averaging of indexes in ties yields multiplicities of some (integer and noninteger) indexes. We consider that this way of processing ties in ranking indexes is not very satisfying and effective. We will show how the new method proposed in this work solves this problem more effectively.

## 4 Invariance under indexing principle (IUIP)

### 4.1 Counter-example for the *F*-distance

This very simple *F*-distance is actually not satisfactory at all because it highly depends on the choice of the indexing of the objects in the reference set, which may yield very different results and conclusions. Based on a very simple counter-example, we show that *F*-distance does not satisfy the principle of invariance under indexing.

As a very simple counter-example, consider the same four distinct objects *A*, *B*, *C*, and *D* as in example 2, and define a new reference set as $Y=\{y_{1}=B,y_{2}=D,y_{3}=A,y_{4}=C\}$. The experts do not change their preference orders, but the reference set is changed here. Therefore, for expert 1 we still have ${\text{Pref}}_{1}≜D≻B≻A≻C$, and ${\text{Pref}}_{2}≜B≻C≻D≻A$ for expert 2. The ranking-indexes expressed in the reference set *Y* are respectively given by $r_{Y,1}=(2,1,3,4)$ because $R_{r_{Y,1}}(Y)=\{y_{2},y_{1},y_{3},y_{4}\}=\{D,B,A,C\}$, and we have $R_{r_{Y,1}}(Y)=R_{r_{X,1}}(X)$. Similarly, one must take $r_{Y,2}=(1,4,2,3)$ because $R_{r_{Y,2}}(Y)=\{y_{1},y_{4},y_{2},y_{3}\}=\{B,C,D,A\}$ and we have in this case $R_{r_{Y,2}}(Y)=R_{r_{X,2}}(X)$. If we calculate the *F*-distance between $r_{Y,1}$ and $r_{Y,2}$ we get (23)

(23) $$F(r_{Y,1},r_{Y,2})=\sum_{i=1}^{4}|r_{Y,1}(i)-r_{Y,2}(i)|=|2-1|+|1-4|+|3-2|+|4-3|=1+3+1+1=6,$$

and (24)

(24) $$\overset{˜}{F}(r_{Y,1},r_{Y,2})=(\frac{2}{n^{2}})\cdot F(r_{Y,1},r_{Y,2})=\frac{2}{16}\cdot 6=0.75.$$

We see that the normalized *F*-distance $\overset{˜}{F}(r_{Y,1},r_{Y,2})=0.75$ between these two ranking-indexes $r_{Y,1}$ and $r_{Y,2}$ is different from $\overset{˜}{F}(r_{X,1},r_{X,2})=1$ obtained in (19). This result and behavior are very counter-intuitive because the rankings for each expert expressed in different reference sets contain exactly the same information about the preference orders, and of course, we have the same rankings because $R_{r_{Y,1}}(Y)=R_{r_{X,1}}(X)$ and $R_{r_{Y,2}}(Y)=R_{r_{X,2}}(X)$. So, there is absolutely no rational reason why the distances between these rankings must be different depending on the reference set chosen (either *X* or *Y*). Our example 2 and this counter-example represent the same ranking information, just expressed in the different reference sets *X* and *Y*, and one sees that we obtain two different results. Which one is correct and makes sense (if any)? Why? This simple counter-example casts in doubt the usefulness of the *F*-distance for applications requiring the measurement of a distance between two rankings. A good distance measure between two rankings must be independent of the choice of the reference set we are working with, which is referred to as invariance under the indexing principle (IUIP). Clearly, Spearman's *F*-distance does not satisfy this important principle.

## 5 A new distance between rankings

To overcome the problem of the non-invariance under indexing of the *F*-distance, we propose a new distance between rankings that satisfies all properties of a metric and satisfies IUIP.

The basic idea of establishing a new distance between rankings is to use all information available in the rankings given by the experts. More precisely, we need to count the different types of preference order in all possible pairwise comparisons between two elements of the reference set under consideration. This is done by calculating the n×n pairwise Preference-Score Matrix (PSM) based on the ranking given by each expert.

By convention, the row index *i* of the PSM corresponds to the index of elements $x_{i}$ on the left side of preference order $x_{i}≻x_{j}$, and the column index *j* of the PSM corresponds to the index of the element $x_{j}$ on the right side of preference order $x_{i}≻x_{j}$. Hence we denote a pairwise Preference-Score Matrix $M_{r_{X}}(X)=[M_{r_{X}}(i,j)]$ where its components $M_{r_{X}}(i,j)$ for i,j=1,2,…,n are defined as (25)

(25) $$M_{r_{X}}(i,j)=\left\{ \begin{array}{cc} 1, & \;\text{if }x_{i}≻x_{j},\\ -1, & \;\text{if }x_{i}≺x_{j},\\ 0, & \;\text{if }x_{i}=x_{j}. \end{array}\right.$$

Note that all components $M_{r_{X}}(i,i)$ (i=1,2,…,n) of the main diagonal of the matrix $M_{r_{X}}$ are always equal to zero. Also, PSM is always an anti-symmetrical matrix by construction because the preference $x_{i}≻x_{j}$ is equivalent to the preference $x_{j}≺x_{i}$. Hence if $x_{i}≻x_{j}$ is true which means $M_{r_{X}}(i,j)=1$ then necessarily $x_{j}≻x_{i}$ is false which means that $x_{j}≺x_{i}$ is true and thus $M_{r_{X}}(j,i)=-1$, and the other way around. Consequently, $M_{r_{X}}{\left(X\right)}^{T}=-M_{r_{X}}(X)$, and $\text{Tr}(M_{r_{X}}(X))=0$.

In example 2, $X=\{x_{1}=A,x_{2}=B,x_{3}=C,x_{4}=D\}$ and the preference order of expert 1 is ${\text{Pref}}_{1}≜D≻B≻A≻C$. Therefore, we have (26)

(26) MrX,1=≻x1=Ax2=Bx3=Cx4=Dx1=A(0−11−1)x2=B101−1x3=C−1−10−1x4=D1110.

The component $M_{r_{X,1}(1,2)}$ of matrix $M_{r_{X,1}}$ equals -1 because the preference $x_{1}≺x_{2}$ is true, or equivalently the preference A≺B is true because in ${\text{Pref}}_{1}≜D≻B≻A≻C$ we have B≻A which is equivalent to the preference A≺B. Other values of components $M_{r_{X,1}(i,j)}$ are obtained from the definition (25).

In example 2 where the reference set is $X=\{x_{1}=A,x_{2}=B,x_{3}=C,x_{4}=D\}$, the preference order of expert 2 is ${\text{Pref}}_{2}≜B≻C≻D≻A$, and we have (27)

(27) MrX,2=≻x1=Ax2=Bx3=Cx4=Dx1=A(0−1−1−1)x2=B1011x3=C1−101x4=D1−1−10.

If we consider our simple counter-example using the reference set $Y=\{y_{1}=B,y_{2}=D,y_{3}=A,y_{4}=C\}$, for expert 1 with ranking ${\text{Pref}}_{1}≜D≻B≻A≻C$ we have now (28)

(28) MrY,1=≻y1=By2=Dy3=Ay4=Cy1=B(0−111)y2=D1011y3=A−1−101y4=C−1−1−10.

If we consider our simple counter-example of Spearman's F-distance using the reference set $Y=\{y_{1}=B,y_{2}=D,y_{3}=A,y_{4}=C\}$, for expert 2 with ranking ${\text{Pref}}_{2}≜B≻C≻D≻A$ we have the following PSM (29)

(29) MrY,2=≻y1=By2=Dy3=Ay4=Cy1=B(0111)y2=D−101−1y3=A−1−10−1y4=C−1110.

At this current stage, we have to find a way to measure the distance between the rankings based on the knowledge of the PSM of each expert. The natural idea is to use directly a distance between PSM matrices. In practice, there are many ways to define the distance between two matrices depending on the choice of a norm for the matrix. We first recall Kemeny's distance and then present our new distance and discuss the differences in their results.

### 5.1 Kemeny's distance

In [16], Kemeny used a particular axiomatic approach to define his distance between preferences orderings. More precisely, if we consider two rankings of *N* objects from which we calculate their associated n×n ordering matrices^4^ $M_{1}=[M_{1}(i,j),i,j=1,\ldots ,n]$ and $M_{2}=[M_{2}(i,j),i,j=1,\ldots ,n]$ respectively, Kemeny's distance between these two rankings are defined as^5^ (30) [16]

(30) $$d_{K}(M_{1},M_{2})=\frac{1}{2}\sum_{i=1}^{n}\sum_{j=1}^{n}|M_{1}(i,j)-M_{2}(i,j)|.$$

Kemeny's distance is a true metric invariant to labeling, and it satisfies our Invariance under the indexing principle (IUIP) because, in his axiomatic approach, he includes IUIP as a requested condition to satisfy (see condition 2 of [17], p. 587).

• Example 2 with $X=\{x_{1}=A,x_{2}=B,x_{3}=C,x_{4}=D\}$
  In our example 2 when working with reference set $X=\{x_{1}=A,x_{2}=B,x_{3}=C,x_{4}=D\}$ and considering the preference orderings ${\text{Pref}}_{1}≜D≻B≻A≻C$ and ${\text{Pref}}_{2}≜B≻C≻D≻A$ we have the ordering matrices (31) and (32)
  (31) $$M_{r_{X,1}}=\left[\begin{array}{cccc} 0 & -1 & 1 & -1\\ 1 & 0 & 1 & -1\\ -1 & -1 & 0 & -1\\ 1 & 1 & 1 & 0 \end{array}\right],$$
  and
  (32) $$M_{r_{X,2}}=\left[\begin{array}{cccc} 0 & -1 & -1 & -1\\ 1 & 0 & 1 & 1\\ 1 & -1 & 0 & 1\\ 1 & -1 & -1 & 0 \end{array}\right].$$
  Applying Kemeny's definition (30) we obtain (33)
  (33) $$d_{K}(M_{r_{X,1}},M_{r_{X,2}})=\frac{1}{2}\sum_{i=1}^{N}\sum_{j=1}^{N}|M_{r_{X,1}}(i,j)-M_{r_{X,2}}(i,j)|=6.$$
  If we want to work with a normalized Kemeny's distance in [0,1], then we need to calculate the maximum Kemeny's distance, which is naturally obtained when the two preference orderings are in total contradiction, that is, for instance, when ${\text{Pref}}_{1}^{\prime }≜A≻B≻C≻D$ and ${\text{Pref}}_{2}^{\prime }≜D≻C≻B≻A$. This corresponds to ordering matrices (34) and (35)
  (34) $$M_{r_{X,1}}^{\prime }=\left[\begin{array}{cccc} 0 & 1 & 1 & 1\\ -1 & 0 & 1 & 1\\ -1 & -1 & 0 & 1\\ -1 & -1 & -1 & 0 \end{array}\right],$$
  and
  (35) $$M_{r_{X,2}}^{\prime }=\left[\begin{array}{cccc} 0 & -1 & -1 & -1\\ 1 & 0 & -1 & -1\\ 1 & 1 & 0 & -1\\ 1 & 1 & 1 & 0 \end{array}\right].$$
  Applying Kemeny's definition (30) we obtain (36)
  (36) $$d_{K}^{\max}=d_{K}(M_{r_{X,1}}^{\prime },M_{r_{X,2}}^{\prime })=\frac{1}{2}\sum_{i=1}^{N}\sum_{j=1}^{N}|M_{r_{X,1}}^{\prime }(i,j)-M_{r_{X,2}}^{\prime }(i,j)|=12.$$
  The normalized Kemeny's distance between preference orderings ${\text{Pref}}_{1}≜D≻B≻A≻C$ and ${\text{Pref}}_{2}≜B≻C≻D≻A$ when working on the reference set $X=\{x_{1}=A,x_{2}=B,x_{3}=C,x_{4}=D\}$ is finally given by (37)
  (37) $${\overset{˜}{d}}_{K}(M_{1},M_{2})=\frac{d_{K}(M_{1},M_{2})}{d_{K}^{\max}}=\frac{6}{12}=0.5$$

• Example 2 with $Y=\{y_{1}=B,y_{2}=D,y_{3}=A,y_{4}=C\}$
  If we consider our example 2 using the reference set $Y=\{y_{1}=B,y_{2}=D,y_{3}=A,y_{4}=C\}$, for expert 1 with ranking ${\text{Pref}}_{1}≜D≻B≻A≻C$ and ${\text{Pref}}_{2}≜B≻C≻D≻A$ we consider now the ordering matrices (38) and (39)
  (38) $$M_{r_{Y,1}}=\left[\begin{array}{cccc} 0 & -1 & 1 & 1\\ 1 & 0 & 1 & 1\\ -1 & -1 & 0 & 1\\ -1 & -1 & -1 & 0 \end{array}\right],$$
  and
  (39) $$M_{r_{Y,2}}=\left[\begin{array}{cccc} 0 & 1 & 1 & 1\\ -1 & 0 & 1 & -1\\ -1 & -1 & 0 & -1\\ -1 & 1 & 1 & 0 \end{array}\right].$$
  Applying Kemeny's definition (30) we obtain (40)
  (40) $$d_{K}(M_{r_{Y,1}},M_{r_{Y,2}})=\frac{1}{2}\sum_{i=1}^{N}\sum_{j=1}^{N}|M_{r_{Y,1}}(i,j)-M_{r_{Y,2}}(i,j)|=6.$$
  If we want to work with a normalized Kemeny's distance in [0,1] then we need to calculate the maximum Kemeny's distance obtained when the two preference orderings are in total contradiction, that is for instance when ${\text{Pref}}_{1}^{\prime }≜A≻B≻C≻D$ and ${\text{Pref}}_{2}^{\prime }≜D≻C≻B≻A$. This corresponds to ordering matrices expressed w.r.t. the reference set $Y=\{y_{1}=B,y_{2}=D,y_{3}=A,y_{4}=C\}$ as follows (41)
  (41) $$M_{r_{Y,1}}^{\prime }=\left[\begin{array}{cccc} 0 & 1 & -1 & 1\\ -1 & 0 & -1 & -1\\ 1 & 1 & 0 & 1\\ -1 & 1 & -1 & 0 \end{array}\right],$$
  and (42)
  (42) $$M_{r_{Y,2}}^{\prime }=\left[\begin{array}{cccc} 0 & -1 & 1 & -1\\ 1 & 0 & 1 & 1\\ -1 & -1 & 0 & -1\\ 1 & -1 & 1 & 0 \end{array}\right].$$
  Applying Kemeny's definition (30) we obtain (43)
  (43) $$d_{K}^{\max}=d_{K}(M_{r_{Y,1}}^{\prime },M_{r_{Y,2}}^{\prime })=\frac{1}{2}\sum_{i=1}^{N}\sum_{j=1}^{N}|M_{r_{Y,1}}^{\prime }(i,j)-M_{r_{Y,2}}^{\prime }(i,j)|=12.$$
  The normalized Kemeny's distance between preference orderings ${\text{Pref}}_{1}≜D≻B≻A≻C$ and ${\text{Pref}}_{2}≜B≻C≻D≻A$ when working on the reference set $Y=\{y_{1}=B,y_{2}=D,y_{3}=A,y_{4}=C\}$ is finally given by (44)
  (44) $${\overset{˜}{d}}_{K}(M_{r_{Y,1}},M_{r_{Y,2}})=\frac{d_{K}(M_{r_{Y,1}},M_{r_{Y,2}})}{d_{K}^{\max}}=\frac{6}{12}=0.5$$

We verify that Kemeny's distance is independent of the reference set chosen (i.e. the indexing, or labeling) for the objects because we have for our example 2 (45):

(45) $${\overset{˜}{d}}_{K}(M_{r_{X,1}},M_{r_{X,2}})={\overset{˜}{d}}_{K}(M_{r_{Y,1}},M_{r_{Y,2}})=0.5$$

Based on this normalized Kemeny's distance ${\overset{˜}{d}}_{K}(M_{r_{X,1}},M_{r_{X,2}})=0.5$ we cannot establish for sure if the two rankings are more similar, or if they are more dissimilar because the normalized Kemeny's distance 0.5 is just in the middle of interval [0,1].

### 5.2 A new ranking distance based on Frobenius' norm

Here, we consider the vectorial space $M_{n}$ of the real square matrices of dimension n×n, and we propose to use the well-known Frobenius' norm, which is one of the most frequent matrix norms used in linear algebra. Frobenius' norm $||M|{|}_{F}$ of a square matrix $M=[M(i,j),i,j=1,\ldots ,n]\in M_{n}$ is defined by (46) [14], [23]

(46) $$||M|{|}_{F}=\sqrt{\sum_{i=1}^{n}\sum_{j=1}^{n}|M(i,j){|}^{2}}=\sqrt{\text{Tr}(M^{T}M)},$$

where $M^{T}$ is the transpose of the matrix ***M***, and Tr(.) is the trace operator for matrix. Based on this norm, the distance between two matrices $M_{1}$ and $M_{2}$ of the same dimensions is simply defined by^6^ (47)

(47) $$d_{F}(M_{1},M_{2})=||M_{1}-M_{2}|{|}_{F}.$$

Theorem *The Frobenius' distance* $d_{F}(M_{1},M_{2})$ *satisfies the invariance under indexing principle.*

Proof Consider a reference set $X=\{x_{1},x_{2},\ldots ,x_{n}\}$ of *n* objects, and another reference set $Y=\{y_{1},y_{2},\ldots ,y_{n}\}$ for these *n* objects, then there is a permutation matrix (U) that transforms *X* into *Y* [22] such that y=Ux, where $y≜{\left[y_{1},y_{2},\ldots ,y_{n}\right]}^{T}$ and $x≜{\left[x_{1},x_{2},\ldots ,x_{n}\right]}^{T}$ [22]. This permutation matrix ***U*** is a unitary orthogonal matrix [23] that verifies $U^{T}U=UU^{T}=I_{n\times n}$, where $I_{n\times n}$ is the n×n identity matrix. If one considers a preference ordering ${\text{Pref}}_{X}$ expressed on the reference set *X* and its corresponding preference ordering ${\text{Pref}}_{Y}$ expressed on the reference set *Y*, the corresponding ordering matrices $M_{X}$ and $M_{Y}$ are similar because they are linked via the orthogonal matrix ***U***. Consequently, we have^7^ (48)

(48) $$M_{Y}=U^{-1}M_{X}U$$

If we consider two ordering matrices $M_{Y,1}=U^{-1}M_{X,1}U$ and $M_{Y,2}=U^{-1}M_{X,2}U$ characterizing two preferences orderings ${\text{Pref}}_{Y,1}$ and ${\text{Pref}}_{Y,2}$ defined on the reference set *Y*, we have (49)

(49) $$M_{Y,1}-M_{Y,2}=U^{-1}M_{X,1}U-U^{-1}M_{X,2}U=U^{-1}(M_{X,1}-M_{X,2})U$$

and its transpose is expressed as (50)

(50) $${\left(M_{Y,1}-M_{Y,2}\right)}^{T}={\left(U^{-1}(M_{X,1}-M_{X,2})U\right)}^{T}=U^{T}{\left(M_{X,1}-M_{X,2}\right)}^{T}{\left(U^{-1}\right)}^{T}$$

Therefore (51),

(51) $${\left(M_{Y,1}-M_{Y,2}\right)}^{T}(M_{Y,1}-M_{Y,2})=U^{T}{\left(M_{X,1}-M_{X,2}\right)}^{T}{\left(U^{-1}\right)}^{T}U^{-1}(M_{X,1}-M_{X,2})U$$

Because $U^{T}U=I_{n\times n}$ (***U*** being a unitary orthogonal matrix), we have $U^{T}=U^{-1}$ and so ${\left(U^{-1}\right)}^{T}={\left(U^{T}\right)}^{T}=U$, so that ${\left(U^{-1}\right)}^{T}U^{-1}=I_{n\times n}$. Therefore the matrix product ${\left(M_{Y,1}-M_{Y,2}\right)}^{T}(M_{Y,1}-M_{Y,2})$ is written as (52)

(52) $${\left(M_{Y,1}-M_{Y,2}\right)}^{T}(M_{Y,1}-M_{Y,2})=U^{T}{\left(M_{X,1}-M_{X,2}\right)}^{T}(M_{X,1}-M_{X,2})U=U^{-1}{\left(M_{X,1}-M_{X,2}\right)}^{T}(M_{X,1}-M_{X,2})U$$

Because the matrices in the trace of a product can be switched without changing the result (which is called the *similarity invariance* of the trace operator) [23] meaning that $\text{Tr}(A)=\text{Tr}(P^{-1}AP)$ for any square matrix ***A*** and any invertible matrix ***P*** of the same dimensions, we always have (53)

(53) $$\text{Tr}({\left(M_{Y,1}-M_{Y,2}\right)}^{T}(M_{Y,1}-M_{Y,2}))=\text{Tr}(U^{-1}{\left(M_{X,1}-M_{X,2}\right)}^{T}(M_{X,1}-M_{X,2})U)=\text{Tr}({\left(M_{X,1}-M_{X,2}\right)}^{T}(M_{X,1}-M_{X,2}))$$

Consequently, we always have (54)

(54) $$d_{F}(M_{Y,1},M_{Y,2})=d_{F}(M_{X,1},M_{X,2})$$

This shows that the Frobenius' distance between two preference orderings characterized by their ordering matrices is invariant under indexing, meaning it is independent of the choice of reference set we work with. This completes the proof of the theorem.

• Example 2 with $X=\{x_{1}=A,x_{2}=B,x_{3}=C,x_{4}=D\}$
  In our example 2 and when working with reference set $X=\{x_{1}=A,x_{2}=B,x_{3}=C,x_{4}=D\}$ we work with PSM $M_{r_{X,1}}$ given in (31) and $M_{r_{X,2}}$ given in (32), and we have (55)
  (55) $$M_{r_{X,1}}-M_{r_{X,2}}=\left[\begin{array}{cccc} 0 & 0 & 2 & 0\\ 0 & 0 & 0 & -2\\ -2 & 0 & 0 & -2\\ 0 & 2 & 2 & 0 \end{array}\right],$$
  and (56)
  (56) $${\left(M_{r_{X,1}}-M_{r_{X,2}}\right)}^{T}=\left[\begin{array}{cccc} 0 & 0 & -2 & 0\\ 0 & 0 & 0 & 2\\ 2 & 0 & 0 & 2\\ 0 & -2 & -2 & 0 \end{array}\right].$$
  Therefore (57),
  (57) $${\left(M_{r_{X,1}}-M_{r_{X,2}}\right)}^{T}(M_{r_{X,1}}-M_{r_{X,2}})=\left[\begin{array}{cccc} 4 & 0 & 0 & 4\\ 0 & 4 & 4 & 0\\ 0 & 4 & 8 & 0\\ 4 & 0 & 0 & 8 \end{array}\right].$$
  Hence (58)
  (58) $$\text{Tr}({\left(M_{r_{X,1}}-M_{r_{X,2}}\right)}^{T}(M_{r_{X,1}}-M_{r_{X,2}}))=4+4+8+8=24$$
  and we finally get (59)
  (59) $$d_{F}(M_{r_{X,1}},M_{r_{X,2}})=\sqrt{24}\approx 4.8990$$
  If we want to work with a normalized distance in [0,1], then we need to calculate the maximum distance that is naturally obtained when the two preference orderings are in total contradiction, that is, for instance, when ${\text{Pref}}_{1}^{\prime }≜A≻B≻C≻D$ and ${\text{Pref}}_{2}^{\prime }≜D≻C≻B≻A$. This corresponds to PSM matrices (60)
  (60) $$M_{r_{X,1}}^{\prime }=\left[\begin{array}{cccc} 0 & 1 & 1 & 1\\ -1 & 0 & 1 & 1\\ -1 & -1 & 0 & 1\\ -1 & -1 & -1 & 0 \end{array}\right],$$
  and (61)
  (61) $$M_{r_{X,2}}^{\prime }=\left[\begin{array}{cccc} 0 & -1 & -1 & -1\\ 1 & 0 & -1 & -1\\ 1 & 1 & 0 & -1\\ 1 & 1 & 1 & 0 \end{array}\right].$$
  Hence (62),
  (62) $$M_{r_{X,1}}^{\prime }-M_{r_{X,2}}^{\prime }=\left[\begin{array}{cccc} 0 & 2 & 2 & 2\\ -2 & 0 & 2 & 2\\ -2 & -2 & 0 & 2\\ -2 & -2 & -2 & 0 \end{array}\right],$$
  and (63)
  (63) $${\left(M_{r_{X,1}}^{\prime }-M_{r_{X,2}}^{\prime }\right)}^{T}=\left[\begin{array}{cccc} 0 & -2 & -2 & -2\\ 2 & 0 & -2 & -2\\ 2 & 2 & 0 & -2\\ 2 & 2 & 2 & 0 \end{array}\right].$$
  Therefore (64),
  (64) $${\left(M_{r_{X,1}}^{\prime }-M_{r_{X,2}}^{\prime }\right)}^{T}(M_{r_{X,1}}^{\prime }-M_{r_{X,2}}^{\prime })=\left[\begin{array}{cccc} 12 & 8 & 0 & -8\\ 8 & 12 & 8 & 0\\ 0 & 8 & 12 & 8\\ -8 & 0 & 8 & 12 \end{array}\right].$$
  Hence (65)
  (65) $$\text{Tr}({\left(M_{r_{X,1}}^{\prime }-M_{r_{X,2}}^{\prime }\right)}^{T}(M_{r_{X,1}}^{\prime }-M_{r_{X,2}}^{\prime }))=12+12+12+12=48,$$
  and we finally get^8^ (66)
  (66) $$d_{F,X}^{\max}=d_{F}(M_{r_{X,1}}^{\prime },M_{r_{X,2}}^{\prime })=\sqrt{48}\approx 6.9282$$
  The normalized Frobenius' distance between preference orderings ${\text{Pref}}_{1}≜D≻B≻A≻C$ and ${\text{Pref}}_{2}≜B≻C≻D≻A$ when working on the reference set $X=\{x_{1}=A,x_{2}=B,x_{3}=C,x_{4}=D\}$ is finally given by (67)
  (67) $${\overset{˜}{d}}_{F}(M_{r_{X,1}},M_{r_{X,2}})=\frac{d_{F}(M_{r_{X,1}},M_{r_{X,2}})}{d_{F,X}^{\max}}\approx 0.7071.$$

• Example 2 with $Y=\{y_{1}=B,y_{2}=D,y_{3}=A,y_{4}=C\}$
  If we consider our example 2 using the reference set $Y=\{y_{1}=B,y_{2}=D,y_{3}=A,y_{4}=C\}$, for expert 1 with ranking ${\text{Pref}}_{1}≜D≻B≻A≻C$ and ${\text{Pref}}_{2}≜B≻C≻D≻A$ we consider now the ordering matrices $M_{r_{Y,1}}$ given by (38), and $M_{r_{Y,2}}$ given by (39). Applying the distance definition (47) we obtain (68)
  (68) $$M_{r_{Y,1}}-M_{r_{Y,2}}=\left[\begin{array}{cccc} 0 & -2 & 0 & 0\\ 2 & 0 & 0 & 2\\ 0 & 0 & 0 & 2\\ 0 & -2 & -2 & 0 \end{array}\right],$$
  and (69)
  (69) $${\left(M_{r_{Y,1}}-M_{r_{Y,2}}\right)}^{T}=\left[\begin{array}{cccc} 0 & 2 & 0 & 0\\ -2 & 0 & 0 & -2\\ 0 & 0 & 0 & -2\\ 0 & 2 & 2 & 0 \end{array}\right].$$
  Therefore (70),
  (70) $${\left(M_{r_{Y,1}}-M_{r_{Y,2}}\right)}^{T}(M_{r_{Y,1}}-M_{r_{Y,2}})=\left[\begin{array}{cccc} 4 & 0 & 0 & 4\\ 0 & 8 & 4 & 0\\ 0 & 4 & 4 & 0\\ 4 & 0 & 0 & 8 \end{array}\right].$$
  Hence (71)
  (71) $$\text{Tr}({\left(M_{r_{Y,1}}-M_{r_{Y,2}}\right)}^{T}(M_{r_{Y,1}}-M_{r_{Y,2}}))=4+8+4+8=24,$$
  and we finally get (72)
  (72) $$d_{F}(M_{r_{Y,1}},M_{r_{Y,2}})=\sqrt{24}\approx 4.8990.$$
  If we want to work with a normalized distance in [0,1], then we need to calculate the maximum distance obtained when the two preference orderings are in total contradiction, that is, for instance, when ${\text{Pref}}_{1}^{\prime }≜A≻B≻C≻D$ and ${\text{Pref}}_{2}^{\prime }≜D≻C≻B≻A$. This corresponds to ordering matrices $M_{r_{Y,1}}^{\prime }$ given by (41) and $M_{r_{Y,2}}^{\prime }$ given by (42) expressed w.r.t. the reference set $Y=\{y_{1}=B,y_{2}=D,y_{3}=A,y_{4}=C\}$. Applying the distance definition (47) we obtain (73)
  (73) $$M_{r_{Y,1}}^{\prime }-M_{r_{Y,2}}^{\prime }=\left[\begin{array}{cccc} 0 & 2 & -2 & 2\\ -2 & 0 & -2 & -2\\ 2 & 2 & 0 & 2\\ -2 & 2 & -2 & 0 \end{array}\right],$$
  and (74)
  (74) $${\left(M_{r_{Y,1}}^{\prime }-M_{r_{Y,2}}^{\prime }\right)}^{T}=\left[\begin{array}{cccc} 0 & -2 & 2 & -2\\ 2 & 0 & 2 & 2\\ -2 & -2 & 0 & -2\\ 2 & -2 & 2 & 0 \end{array}\right],$$
  and (75)
  (75) $${\left(M_{r_{Y,1}}^{\prime }-M_{r_{Y,2}}^{\prime }\right)}^{T}(M_{r_{Y,1}}^{\prime }-M_{r_{Y,2}}^{\prime })=\left[\begin{array}{cccc} 12 & 0 & 8 & 8\\ 0 & 12 & -8 & 8\\ 8 & -8 & 12 & 0\\ 8 & 8 & 0 & 12 \end{array}\right].$$
  Hence (76)
  (76) $$\text{Tr}({\left(M_{r_{Y,1}}^{\prime }-M_{r_{Y,2}}^{\prime }\right)}^{T}(M_{r_{Y,1}}^{\prime }-M_{r_{Y,2}}^{\prime }))=12+12+12+12=48,$$
  and we finally get (77)
  (77) $$d_{F,Y}^{\max}=d_{F}(M_{r_{Y,1}}^{\prime },M_{r_{Y,2}}^{\prime })=\sqrt{48}\approx 6.9282.$$
  The normalized Frobenius' distance between preference orderings ${\text{Pref}}_{1}≜D≻B≻A≻C$ and ${\text{Pref}}_{2}≜B≻C≻D≻A$ when working on the reference set $Y=\{y_{1}=B,y_{2}=D,y_{3}=A,y_{4}=C\}$ is finally given by (78)
  (78) $$\overset{˜}{d}(M_{r_{Y,1}},M_{r_{Y,2}})=\frac{d_{F}(M_{r_{Y,1}},M_{r_{Y,2}})}{d_{F,Y}^{\max}}\approx 0.7071.$$

We have verified that this new ranking distance based on the Frobenius' norm is independent of the reference set chosen (i.e., the indexing, or labeling) for the objects because we have, for our example 2 (79)

(79) $${\overset{˜}{d}}_{F}(M_{r_{X,1}},M_{r_{X,2}})={\overset{˜}{d}}_{F}(M_{r_{Y,1}},M_{r_{Y,2}})\approx 0.7071.$$

Remark 1 It is worth to check that the unitary orthogonal matrix ***U*** for the permutation from the reference set $X=\{x_{1}=A,x_{2}=B,x_{3}=C,x_{4}=D\}$ to the reference set $Y=\{y_{1}=B,y_{2}=D,y_{3}=A,y_{4}=C\}$ is given by (80)

(80) $$U=\left[\begin{array}{cccc} 0 & 0 & 1 & 0\\ 1 & 0 & 0 & 0\\ 0 & 0 & 0 & 1\\ 0 & 1 & 0 & 0 \end{array}\right].$$

As mentioned in the proof of the Theorem, we can verify that $U^{T}U=UU^{T}=I_{4\times 4}$, and the equalities (81) and (82) hold.

(81) $$M_{r_{Y,1}}=U^{T}M_{r_{X,1}}U,$$

(82) $$M_{r_{Y,2}}=U^{T}M_{r_{X,2}}U.$$

Remark 2 In our example 2, it is interesting to observe that normalized Kemeny's distance and normalized Frobenius' distance between the two rankings of example 2 provide different interpretations about these rankings. Based on normalized Kemeny's distance ${\overset{˜}{d}}_{K}(M_{r_{X,1}},M_{r_{X,2}})=0.5$, it is clear that we cannot assert for sure if the two preference orderings ${\text{Pref}}_{1}≜D≻B≻A≻C$ and ${\text{Pref}}_{2}≜B≻C≻D≻A$ are more similar than dissimilar because the distance 0.5 we get is in the middle of interval [0,1]. However, based on the normalized Frobenius' distance ${\overset{˜}{d}}_{F}(M_{r_{X,1}},M_{r_{X,2}})=0.7071$, we can clearly infer that ${\text{Pref}}_{1}$ and ${\text{Pref}}_{2}$ are more dissimilar than similar because their $d_{F}=0.7071$ distance is closer to 1, than to 0. Which interpretation is correct and makes sense? To answer this question, we must examine the relative consistencies and inconsistencies in ${\text{Pref}}_{1}$ and ${\text{Pref}}_{2}$ orderings, which are summarized in Table 1, Table 2.

**Table 1:** Relative consistencies in Pref_1_ and Pref_2_.

Pref_1_	Pref_2_
*D* ≻ ≻*A*	*D* ≻ *A*
*B* ≻ *A*	*B* ≻ ≻≻*A*
*B* ≻ ≻*C*	*B* ≻ *C*

**Table 2:** Inconsistencies in Pref_1_ and Pref_2_.

Pref_1_	Pref_2_
*D* ≻ *B*	*B* ≻ ≻*D*
*D* ≻ ≻≻*C*	*C* ≻ *D*
*A* ≻ *C*	*C* ≻ ≻*A*

In the Table 1, Table 2, the double ≻≻ notation indicates that there is one object between the left-object side of ≻≻ and its right-object side. For instance, in Table 1, D≻≻A means that we have “D≻some object≻A”. Similarly, the triple ≻≻≻ notation indicates that there are two objects in between.

Based on the Table 1, Table 2, one could argue that preference orderings include three relative consistencies and three inconsistencies, and so we may consider there is no reason to establish that they are more similar than dissimilar. This is what Kemeny's distance tells us with ${\overset{˜}{d}}_{K}(M_{r_{X,1}},M_{r_{X,2}})=0.5$. We think that this reasoning is disputable because we note that the relative consistencies of Table 1 are different. For instance, in Table 1 we have B≻A for ${\text{Pref}}_{1}$, whereas B≻≻≻A for ${\text{Pref}}_{2}$, etc. So, we think it is more reasonable to consider ${\text{Pref}}_{1}$ and ${\text{Pref}}_{2}$ as more dissimilar than similar, and this is what expresses the Frobenius' distance ${\overset{˜}{d}}_{F}(M_{r_{X,1}},M_{r_{X,2}})=0.7071$.

### 5.3 On Kemeny's axiomatic

We recall the four axioms used by Kemeny's to justify his distance (see [16], Chap. 2).

• **Axiom 1**:
  **–** Axiom 1.1: d(A,B)≥0, and inequality holds if and only if *A* and *B* are same ranking.
  **–** Axiom 1.2: d(A,B)=d(B,A).
  **–** Axiom 1.3: d(A,B)+d(B,C)≥d(A,C), and the equality holds if and only if the ranking *B* is between *A* and *C*.
• **Axiom 2**: If $A^{\prime }$ results of *A* by a permutation of objects, and $B^{\prime }$ results of *B* by the same permutation of objects, then $d(A^{\prime },B^{\prime })=d(A,B)$.
• **Axiom 3**: If two rankings *A* and *B* agree except for a set *S* of *k* elements, which is a segment of both, then d(A,B) may be computed as if these *k* objects where the only objects being ranked.
• **Axiom 4**: The minimum positive distance is 1.

Axiom 1 stipulates that the distance must be a true metric, and axiom 2 corresponds to the invariance under the indexing principle. Axioms 1 & 2 are good natural axioms for establishing a distance between rankings. To verify Axiom 1.3, Kemeny's needs to choose a notion of “betweenness”. The definition of a distance based on a norm of a matrix is more general and mathematically well defined. This is why we prefer to use the Frobenius' norm of a matrix for establishing the Frobenius' distance between rankings in this study.

Kemeny's Axiom 3 stipulates that if two rankings are in complete agreement at the beginning and at the end of the list and differ only in the middle, then the distance does not change after deleting both the first and the last objects to be ranked [1]. This axiom 3 is not so intuitive in our opinion but is rather a consequence of working with PSM. Obviously, Frobenius' distance $d_{F}$ satisfies Kemeny's axiom 3 because the matrix $M=M_{1}-M_{2}$ will be a square matrix with all its bordering elements equal to zero because the first elements and the last elements of the rankings are the same for the conditions of rankings expressed in Axiom 3. Consequently, the distance result $d_{F}$ will depend only of the non-zero elements of $M=M_{1}-M_{2}$ (i.e. the elements of its “interior” sub-matrix ${\text{M}}_{\text{int}}$). For instance, if one considers four objects with preferences ${\text{Pref}}_{1}=A≻B≻C≻D$ and ${\text{Pref}}_{2}=A≻C≻B≻D$ satisfying conditions of Axiom 3, then we have the PSM (83)

(83) $$M_{1}=\left[\begin{array}{cccc} 0 & 1 & 1 & 1\\ -1 & 0 & 1 & 1\\ -1 & -1 & 0 & 1\\ -1 & -1 & -1 & 0 \end{array}\right],\;M_{2}=\left[\begin{array}{cccc} 0 & 1 & 1 & 1\\ -1 & 0 & -1 & 1\\ -1 & 1 & 0 & 1\\ -1 & -1 & -1 & 0 \end{array}\right].$$

Hence, $M_{1}-M_{2}$ is the zero-border matrix equal to (84)

(84) $$M=M_{1}-M_{2}=\left[\begin{array}{cccc} 0 & 0 & 0 & 0\\ 0 & 0 & 2 & 0\\ 0 & -2 & 0 & 0\\ 0 & 0 & 0 & 0 \end{array}\right]=\left[\begin{array}{ccc} 0 & \ldots & 0\\ \vdots & M_{\text{int}} & \vdots \\ 0 & \ldots & 0 \end{array}\right],$$

where the interior sub-matrix $M_{\text{int}}$ is (85)

(85) $$M_{\text{int}}=\left[\begin{array}{cc} 0 & 2\\ -2 & 0 \end{array}\right].$$

Therefore (86),

(86) $$M^{T}M=\left[\begin{array}{ccc} 0 & \ldots & 0\\ \vdots & M_{\text{int}}^{T}M_{\text{int}} & \vdots \\ 0 & \ldots & 0 \end{array}\right].$$

Clearly, $\text{Tr}(M^{T}M)=\text{Tr}(M_{\text{int}}^{T}M_{\text{int}})$, and Frobenius' distance is $d_{F}(M_{1},M_{2})=\sqrt{\text{Tr}(M^{T}M)}=\sqrt{\text{Tr}(M_{\text{int}}^{T}M_{\text{int}})}$. This proves that the Frobenius' distance between $M_{1}$ and $M_{2}$ does not change after deleting both the first and the last objects to be ranked because it depends only on interior sub-matrix $M_{\text{int}}$ which is nothing but the PSM of objects in the middle of rankings that have swapped.

**Figure 1:**
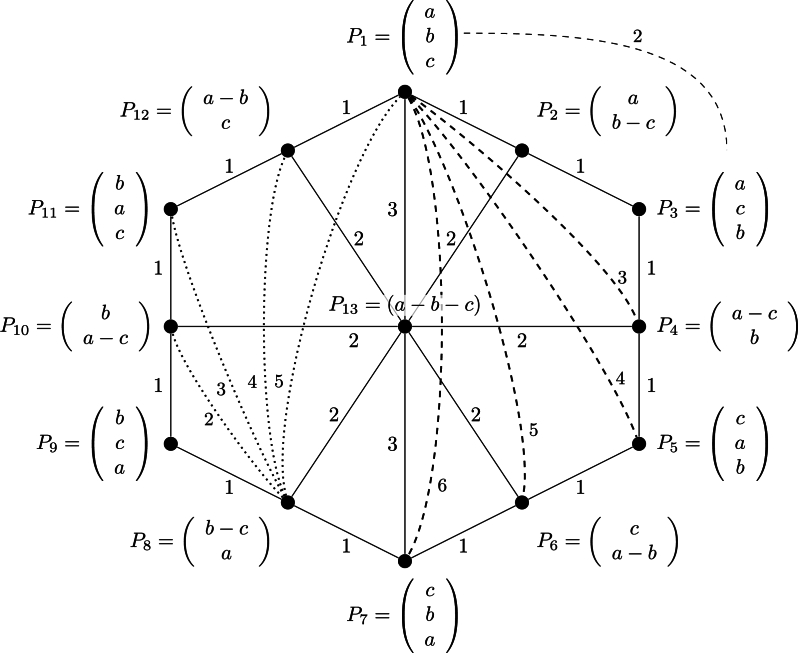
Kemeny's distances for 3 rankings.

As written by Kemeny himself in [16] (p. 10), the axiom 4 is “in the nature of a convention”. This axiom 4 has been chosen to fit with Kemeny's distance definition, but it is actually arbitrary and disputable. It is worth noting that this minimal Kemeny's positive distance of 1 is obtained only between a strict (i.e., proper) ranking and a tied ranking as shown by Kemeny's in his example for the 3-objects case (see Fig. 2 of [16], p. 17, and next in this paper on Fig. 1). We can also justify the Frobenius' distance between rankings by modifying the arbitrary Kemeny's axiom 4 in order to fit with the Frobenius' distance definition as well, and thus requiring that the minimum positive distance is $\sqrt{2}$ (because $\sqrt{2}$ is the minimum positive Frobenius' distance between the simple preference A≻B and the tie A=B). This would not be more arbitrary than the choice made by Kemeny for his axiom 4. Or if we prefer, we can scale (i.e. divide) the Frobenius's distance by the factor $\sqrt{2}$ to satisfy Kemeny's axiom 4, and we can work with $\frac{1}{\sqrt{2}}d_{F}(M_{1},M_{2})$ instead of Kemeny's distance without violating Kemeny's axiomatic.

Consequently, Kemeny's statement about the unicity of his distance verifying his axiomatic is wrong because the ($\sqrt{2}$-scaled) Frobenius' distance also satisfies his axiomatic. The justification of Frobenius' distance between rankings has the same axiomatic strength as Kemeny's approach, and it cannot be disputed or discarded based on Kemeny's axiomatic argumentation.

### 5.4 Comparison of Frobenius' distance with Kemeny's distance

**Comparison 1.** We use Kemeny's example [16] (p. 17) for ranking three objects. Kemeny's result is shown in Fig. 1. We recall the equivalence between Kemeny's notation for preference ordering and our notations in Table 3.

**Table 3:** Equivalence between Kemeny's notation and ours.

Preference #	Kemeny's notation	our notation
*P*_1_	$\left(\begin{array}{c} a\\ b\\ c \end{array}\right)$	*a* ≻ *b* ≻ *c*
*P*_2_	$\left(\begin{array}{c} a\\ b-c \end{array}\right)$	*a* ≻ (*b* = *c*)
*P*_3_	$\left(\begin{array}{c} a\\ c\\ b \end{array}\right)$	*a* ≻ *c* ≻ *b*
*P*_4_	$\left(\begin{array}{c} a-c\\ b \end{array}\right)$	(*a* = *c*)≻*b*
*P*_5_	$\left(\begin{array}{c} c\\ a\\ b \end{array}\right)$	*c* ≻ *a* ≻ *b*
*P*_6_	$\left(\begin{array}{c} c\\ a-b \end{array}\right)$	*c* ≻ (*a* = *b*)
*P*_7_	$\left(\begin{array}{c} c\\ b\\ a \end{array}\right)$	*c* ≻ *b* ≻ *a*
*P*_8_	$\left(\begin{array}{c} b-c\\ a \end{array}\right)$	(*b* = *c*)≻*a*
*P*_9_	$\left(\begin{array}{c} b\\ c\\ a \end{array}\right)$	*b* ≻ *c* ≻ *a*
*P*_10_	$\left(\begin{array}{c} b\\ a-c \end{array}\right)$	*b* ≻ (*a* = *c*)
*P*_11_	$\left(\begin{array}{c} b\\ a\\ c \end{array}\right)$	*b* ≻ *a* ≻ *c*
*P*_12_	$\left(\begin{array}{c} a-b\\ c \end{array}\right)$	(*a* = *b*)≻*c*
*P*_13_	$\left(\begin{array}{c} a-b-c \end{array}\right)$	*a* = *b* = *c*

For convenience and for comparison with Frobenius' distances in our study, additional links from point (i.e. preference) $P_{1}$ to preferences $P_{4}$, $P_{5}$, $P_{6}$ and $P_{7}$ with their Kemeny's distances have been also included on Fig. 1, as well as distances from $P_{2}$ to $P_{4}$, $P_{5}$, $P_{6}$ and $P_{7}$. For symmetrical reasons, other links between other preferences are not necessary to be shown.

**Figure 2:**
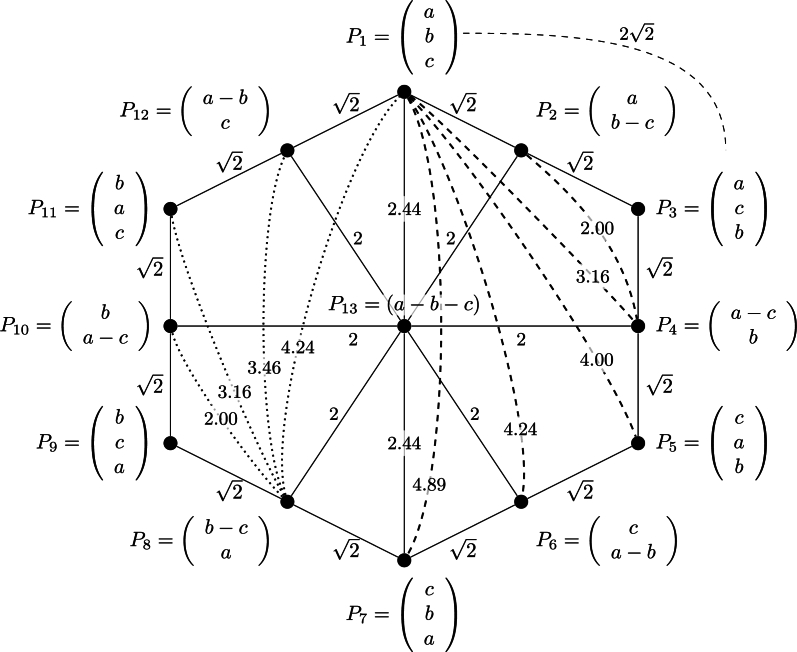
Frobenius' distances for 3 rankings.

The same representation based on Frobenius' distance is shown on Fig. 2.

The comparison of Figure 1, Figure 2 shows the differences between Kemeny's and Frobenius' distances. What is remarkable is that Kemeny's distance from one point to another always equals the sum of the values of the path between the two points, whose sum is minimal. For instance, $d_{K}(P_{1},P_{13})=d_{K}(P_{1},P_{2})+d_{K}(P_{2},P_{13})=1+2=3$, which means that the triangular inequality condition is an equality condition. Using Frobenius' distance, the triangular inequality holds because $d_{F}(P_{1},P_{13})=2.44<d_{F}(P_{1},P_{2})+d_{F}(P_{2},P_{13})=\sqrt{2}+2=3.4142$. Similarly, $d_{K}(P_{2},P_{6})=d_{K}(P_{2},P_{13})+d_{K}(P_{13},P_{6})=2+2=4$, whereas $d_{F}(P_{2},P_{6})=3.46<d_{F}(P_{2},P_{13})+d_{F}(P_{13},P_{6})=2+2=4$. Other verifications can be easily done using most distance values shown in Figure 1, Figure 2. We think that this behavior of Frobenius' distance is more reasonable than Kemeny's distance.

**Figure 3:**
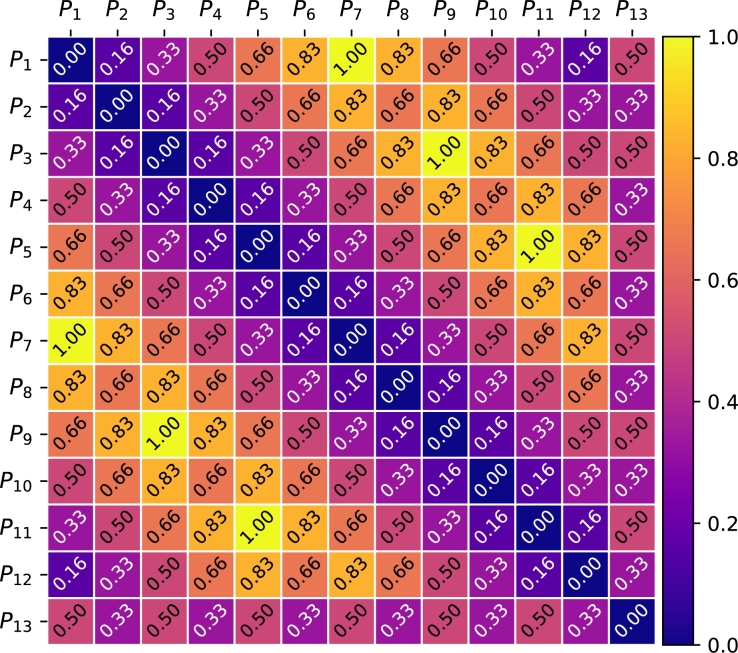
All possible normalized Kemeny's distances for 3 rankings.

**Figure 4:**
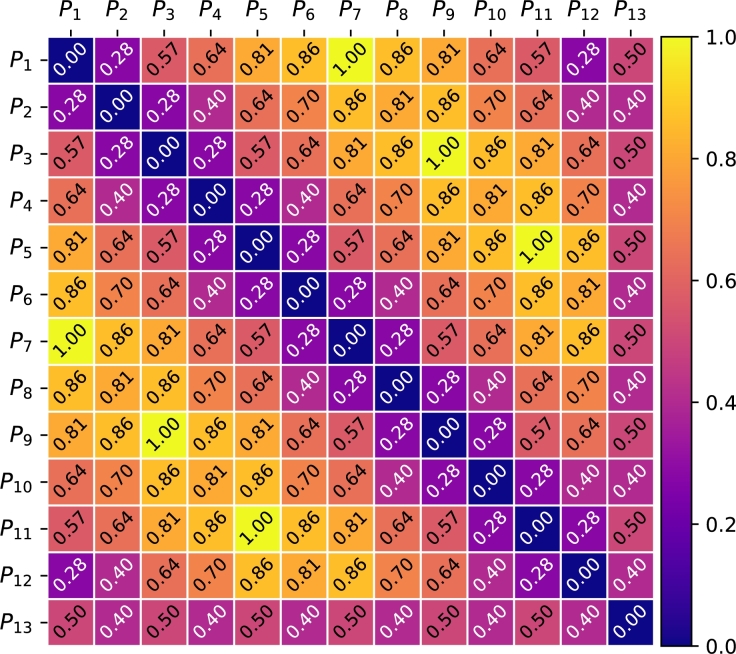
All possible normalized Frobenius' distances for 3 rankings.

Note that “normalized figures” can be obtained by dividing the $d_{K}$ values of each link of Fig. 1 by $d_{K}^{\max}=6$, and by dividing the $d_{F}$ values of each link of Fig. 2 by $d_{F}^{\max}\approx 4.8990$. The normalized distances (Kemeny's and Frobenius') between all possible rankings of three objects are shown in Figure 3, Figure 4 for convenience.

**Comparison 2.** To assess the differences between normalized Kemeny's distance ${\overset{˜}{d}}_{K}(.,.)$ and the normalized Frobenius' distance ${\overset{˜}{d}}_{F}(.,.)$, we make a comparative analysis when considering the strict preference ordering ${\text{Pref}}_{1}≜A≻B≻C≻D$ with respect to all possible (i.e. 24) strict^9^ preferences ${\text{Pref}}_{2}$ generated from all possible permutations of 4 elements. The results are listed in Table 4.

**Table 4:** Comparison of ${\overset{˜}{d}}_{K}(.,.)$ and ${\overset{˜}{d}}_{F}(.,.)$ between preference orderings Pref_1_ and Pref_2_.

Pref_1_	Pref_2_	${\overset{˜}{d}}_{K}(.,.)$	${\overset{˜}{d}}_{F}(.,.)$
*A* ≻ *B* ≻ *C* ≻ *D*	*A* ≻ *B* ≻ *C* ≻ *D*	0	0
*A* ≻ *B* ≻ *C* ≻ *D*	*A* ≻ *B* ≻ *D* ≻ *C*	0.1667	0.4082
*A* ≻ *B* ≻ *C* ≻ *D*	*A* ≻ *C* ≻ *B* ≻ *D*	0.1667	0.4082
*A* ≻ *B* ≻ *C* ≻ *D*	*A* ≻ *C* ≻ *D* ≻ *B*	0.3333	0.5774
*A* ≻ *B* ≻ *C* ≻ *D*	*A* ≻ *D* ≻ *B* ≻ *C*	0.3333	0.5774
*A* ≻ *B* ≻ *C* ≻ *D*	*A* ≻ *D* ≻ *C* ≻ *B*	0.5000	0.7071

*A* ≻ *B* ≻ *C* ≻ *D*	*B* ≻ *A* ≻ *C* ≻ *D*	0.1667	0.4082
*A* ≻ *B* ≻ *C* ≻ *D*	*B* ≻ *A* ≻ *D* ≻ *C*	0.3333	0.5774
*A* ≻ *B* ≻ *C* ≻ *D*	*B* ≻ *C* ≻ *A* ≻ *D*	0.3333	0.5774
*A* ≻ *B* ≻ *C* ≻ *D*	*B* ≻ *C* ≻ *D* ≻ *A*	0.5000	0.7071
*A* ≻ *B* ≻ *C* ≻ *D*	*B* ≻ *D* ≻ *A* ≻ *C*	0.5000	0.7071
*A* ≻ *B* ≻ *C* ≻ *D*	*B* ≻ *D* ≻ *C* ≻ *A*	0.6667	0.8165

*A* ≻ *B* ≻ *C* ≻ *D*	*C* ≻ *A* ≻ *B* ≻ *D*	0.3333	0.5774
*A* ≻ *B* ≻ *C* ≻ *D*	*C* ≻ *A* ≻ *D* ≻ *B*	0.5000	0.7071
*A* ≻ *B* ≻ *C* ≻ *D*	*C* ≻ *B* ≻ *A* ≻ *D*	0.5000	0.7071
*A* ≻ *B* ≻ *C* ≻ *D*	*C* ≻ *B* ≻ *D* ≻ *A*	0.6667	0.8165
*A* ≻ *B* ≻ *C* ≻ *D*	*C* ≻ *D* ≻ *A* ≻ *B*	0.6667	0.8165
*A* ≻ *B* ≻ *C* ≻ *D*	*C* ≻ *D* ≻ *B* ≻ *A*	0.8333	0.9129

*A* ≻ *B* ≻ *C* ≻ *D*	*D* ≻ *A* ≻ *B* ≻ *C*	0.5000	0.7071
*A* ≻ *B* ≻ *C* ≻ *D*	*D* ≻ *A* ≻ *C* ≻ *B*	0.6667	0.8165
*A* ≻ *B* ≻ *C* ≻ *D*	*D* ≻ *B* ≻ *A* ≻ *C*	0.6667	0.8165
*A* ≻ *B* ≻ *C* ≻ *D*	*D* ≻ *B* ≻ *C* ≻ *A*	0.8333	0.9129
*A* ≻ *B* ≻ *C* ≻ *D*	*D* ≻ *C* ≻ *A* ≻ *B*	0.8333	0.9129
*A* ≻ *B* ≻ *C* ≻ *D*	*D* ≻ *C* ≻ *B* ≻ *A*	1	1

From Table 4, we observe the different distances we obtain with Kemeny's distance and Frobenius' distance except in the total consistency case for which ${\overset{˜}{d}}_{K}(M_{1},M_{2})={\overset{˜}{d}}_{F}(M_{1},M_{2})=0$ and in the total contradictions case for which ${\overset{˜}{d}}_{K}(M_{1},M_{2})={\overset{˜}{d}}_{F}(M_{1},M_{2})=1$. This result makes sense, and it is naturally expected. We observe also that we always have ${\overset{˜}{d}}_{K}(M_{1},M_{2})\leq {\overset{˜}{d}}_{F}(M_{1},M_{2})$. The choice between Kemeny's distance and Frobenius' distance for measuring the distance between rankings is not clear at this stage of our study for the applications because both distances $d_{K}$ and $d_{F}$ verify Kemeny's axioms 1, 2 & 3, and they differ only in the arbitrary convention for the axiom 4. Only evaluation of these distances on real applications may help to choose between $d_{K}$ and $d_{F}$ distances in practice.

### 5.5 Dealing with ties

Because of how the proposed approach handles the ranking thanks to the Preference-Score Matrix, the ties do not create any problems here. When some alternatives in the ranking are in ties, the Preference-Score Matrix will be filled with 0 on specific positions when the alternatives are equal. Then, the procedure remains the same. Distances between rankings, including ties, are shown in Figure 1, Figure 2 for the three objects ranking example.

### 5.6 Extension with weights

It is also possible to extend this approach with positive importance weights to calculate weighted distance, which could be useful in some cases. For example, to calculate the distance between two ranking of *n* values, we first have to define a weights vector ***w*** with (87)

(87) $$w={\left[w_{1},w_{2},\ldots ,w_{n}\right]}^{T}.$$

The choice of importance weights vector ***w*** is generally left to the user. For example, weights vector (88) was successfully used in weighted similarity correlation coefficient [28], which, however, does not follow IUIP. The sum of this progression could be calculated according to (89).

(88) $$w^{\left(2\right)}={\left[2^{-1},2^{-2},\ldots ,2^{-n}\right]}^{T},$$

(89) $$\sum_{i=1}^{n}w^{\left(2\right)}(i)=2^{-1}+2^{-2}+\ldots +2^{-n}=1-2^{-n}.$$

By convention, $w_{1}$ is the weight for the best-preferred object, $w_{2}$ is the weight for the 2nd best-preferred object, etc. The sum of the weights is not necessarily equal to one. This does not matter if we work with normalized weighted Frobenius' distance.

Suppose we have a chosen a reference set *X* of *n* objects and two preferences orderings ${\text{Pref}}_{1}$ and ${\text{Pref}}_{2}$ from which the ordering matrices $M_{X,1}$ and $M_{X,2}$ are derived. From $M_{X,1}$ and $M_{X,2}$ we calculate the scoring vector $s_{X,1}$ and $s_{X,2}$ defined by (90)

(90) $$s_{X,1}=\left[\begin{array}{c} \sum_{j=1}^{n}M_{X,1}(1,j)\\ \vdots \\ \sum_{j=1}^{n}M_{X,1}(n,j) \end{array}\right],$$

and (91)

(91) $$s_{X,2}=\left[\begin{array}{c} \sum_{j=1}^{n}M_{X,2}(1,j)\\ \vdots \\ \sum_{j=1}^{n}M_{X,2}(n,j) \end{array}\right].$$

We can sort each value of score vector $s_{X,1}$ and $s_{X,2}$ by their *decreasing sorting* to obtain the respective sorted vectors $s_{X,1}^{\text{sorted}}$ and $s_{X,2}^{\text{sorted}}$ in order to identify the position of the weight $w_{i}$ we must assign to each value. This is called the *weighting assignment of score values*, which is characterized by the weighting vectors $w_{X,1}$ and $w_{X,2}$. Because the score vector $s_{X,1}$ can always be obtained from the (decreasing) sorted score vector $s_{X,1}^{\text{sorted}}$ by a unitary permutation matrix $V_{X,1}$ such that $s_{X,1}=V_{X,1}s_{X,1}^{\text{sorted}}$, and similarly because $s_{X,2}=V_{X,2}s_{X,2}^{\text{sorted}}$ with a n×n unitary permutation matrix $V_{X,2}$, the weighting vectors $w_{X,1}$ and $w_{X,2}$ are obtained respectively by (92) and (93)

(92) $$w_{X,1}=V_{X,1}w,$$

(93) $$w_{X,2}=V_{X,2}w.$$

From weights vectors $w_{X,1}$ and $w_{X,2}$ we build weighting diagonal matrices $W_{X,1}$ and $W_{X,2}$ defined by (94) and (95)

(94) $$W_{X,1}=diag(w_{X,1}),$$

(95) $$W_{X,2}=diag(w_{X,2}).$$

The notation $diag(w_{X,k})$ for k=1,2 represents the square diagonal matrix having its main diagonal terms equal to the elements of vector $w_{X,k}$, and all its non-diagonal elements equal zero. The weighted Frobenius' distance between rankings is simply defined by the distance between weighted ordering matrices $W_{X,1}M_{X,1}$ and $W_{X,2}M_{X,2}$ which is mathematically expressed as (96)

(96) $$d_{F,w}(M_{X,1},M_{X,2})={\left[\text{Tr}({\left(W_{X,1}M_{X,1}-W_{X,2}M_{X,2}\right)}^{T}(W_{X,1}M_{X,1}-W_{X,2}M_{X,2}))\right]}^{\frac{1}{2}}.$$

It can be easily verified that this weighted Frobenius' distance is also invariant to indexing. This verification is left to the reader.^10^

If one wants to use the Frobenius' distance with the normalized weights, it is possible either by using normalized weights $\overset{˜}{w}$, where ${\overset{˜}{w}}_{i}=\frac{w_{i}}{\sum_{i}^{n}w_{i}}$ or using the Equation (97), which utilizes the sum of the non-normalized weights to normalize the distance after the weighting. Both methods are equivalent and provide same results.

(97) $$d_{F,\overset{˜}{w}}(M_{X,1},M_{X,2})={\left[\text{Tr}({\left(W_{X,1}M_{X,1}-W_{X,2}M_{X,2}\right)}^{T}(W_{X,1}M_{X,1}-W_{X,2}M_{X,2}))\right]}^{\frac{1}{2}}/\sum_{i=1}^{n}w(i)$$

In particular case of using weights $w^{\left(2\right)}$ (88) the sum can be expressed as (89), therefore the Equation (97) has the form of (98).

(98) $$d_{F,\overset{˜}{w}}(M_{X,1},M_{X,2})={\left[\text{Tr}({\left(W_{X,1}M_{X,1}-W_{X,2}M_{X,2}\right)}^{T}(W_{X,1}M_{X,1}-W_{X,2}M_{X,2}))\right]}^{\frac{1}{2}}/(1-2^{-n})$$

### 5.7 Example 3: $d_{F,w}$ calculation between rankings

To show in detail how to calculate the weighted Frobenius' distance between rankings, we consider for simplicity two preference orderings of three objects *A*, *B* and *C* chosen as ${\text{Pref}}_{1}≜A≻B≻C$ and ${\text{Pref}}_{2}≜A≻C≻B$. We show how the calculations are done based on the two reference sets $X=\{x_{1}=A,x_{2}=B,x_{3}=C\}$ and $Y=\{y_{1}=B,y_{2}=C,y_{3}=A\}$ to calculate $d_{F,w}(M_{X,1},M_{X,2})$ and $d_{F,w}(M_{Y,1},M_{Y,2})$.

**Case 1**: Using reference set $X=\{x_{1}=A,x_{2}=B,x_{3}=C\}$

With the reference set $X=\{x_{1}=A,x_{2}=B,x_{3}=C\}$ and for the preferences ${\text{Pref}}_{1}≜A≻B≻C$ and ${\text{Pref}}_{2}≜A≻C≻B$, we obtain the following ordering matrices (i.e. PSM) $M_{X,1}$ (99) and $M_{X,2}$ (100) with their scores $s_{X,1}$ and $s_{X,2}$

(99) $$M_{X,1}=\left[\begin{array}{ccc} 0 & 1 & 1\\ -1 & 0 & 1\\ -1 & -1 & 0 \end{array}\right],\;\text{and}\;s_{X,1}=\left[\begin{array}{c} 2\\ 0\\ -2 \end{array}\right],$$

(100) $$M_{X,2}=\left[\begin{array}{ccc} 0 & 1 & 1\\ -1 & 0 & -1\\ -1 & 1 & 0 \end{array}\right],\;\text{and}\;s_{X,2}=\left[\begin{array}{c} 2\\ -2\\ 0 \end{array}\right].$$

Sorting elements of score vectors $s_{X,1}$ and $s_{X,2}$ by descending order yields^11^ (101)

(101) $$s_{X,1}^{sorted}=\left[\begin{array}{c} 2\\ 0\\ -2 \end{array}\right],\;\text{and}\;s_{X,2}^{sorted}=\left[\begin{array}{c} 2\\ 0\\ -2 \end{array}\right].$$

The unitary permutation matrices $V_{X,1}$ and $V_{X,2}$ are respectively given by (102) and (103)

(102) $$V_{X,1}=\left[\begin{array}{ccc} 1 & 0 & 0\\ 0 & 1 & 0\\ 0 & 0 & 1 \end{array}\right]=I_{3\times 3},$$

and

(103) $$V_{X,2}=\left[\begin{array}{ccc} 1 & 0 & 0\\ 0 & 0 & 1\\ 0 & 1 & 0 \end{array}\right].$$

Suppose that the importance weights vector is chosen as in (88) with n=3, that is (104)

(104) $$w={\left[w_{1}=1/2,w_{2}=1/4,w_{3}=1/8\right]}^{T}.$$

The weighting vectors $w_{X,1}$ and $w_{X,2}$ are given by formulas (92) - (93), and we get (105) and (106) respectively:

(105) $$w_{X,1}=V_{X,1}w=I_{3\times 3}w=w=\left[\begin{array}{c} 1/2\\ 1/4\\ 1/8 \end{array}\right],$$

(106) $$w_{X,2}=V_{X,2}w=\left[\begin{array}{ccc} 1 & 0 & 0\\ 0 & 0 & 1\\ 0 & 1 & 0 \end{array}\right]\left[\begin{array}{c} 1/2\\ 1/4\\ 1/8 \end{array}\right]=\left[\begin{array}{c} 1/2\\ 1/8\\ 1/4 \end{array}\right].$$

Based on $w_{X,1}$ and $w_{X,2}$ one gets the weighting matrices (107) and (108):

(107) $$W_{X,1}=diag(w_{X,1})=\left[\begin{array}{ccc} w_{1} & 0 & 0\\ 0 & w_{2} & 0\\ 0 & 0 & w_{3} \end{array}\right]=\left[\begin{array}{ccc} 1/2 & 0 & 0\\ 0 & 1/4 & 0\\ 0 & 0 & 1/8 \end{array}\right],$$

(108) $$W_{X,2}=diag(w_{X,2})=\left[\begin{array}{ccc} w_{1} & 0 & 0\\ 0 & w_{3} & 0\\ 0 & 0 & w_{2} \end{array}\right]=\left[\begin{array}{ccc} 1/2 & 0 & 0\\ 0 & 1/8 & 0\\ 0 & 0 & 1/4 \end{array}\right].$$

The matrix products $W_{X,1}M_{X,1}$ and $W_{X,2}M_{X,2}$ are (109) and (110) respectively:

(109) $$W_{X,1}M_{X,1}=\left[\begin{array}{ccc} 0 & 1/2 & 1/2\\ -1/4 & 0 & 1/4\\ -1/8 & -1/8 & 0 \end{array}\right],$$

and

(110) $$W_{X,2}M_{X,2}=\left[\begin{array}{ccc} 0 & 1/2 & 1/2\\ -1/8 & 0 & -1/8\\ -1/4 & 1/4 & 0 \end{array}\right].$$

Their difference is (111)

(111) $$W_{X,1}M_{X,1}-W_{X,2}M_{X,2}=\left[\begin{array}{ccc} 0 & 0 & 0\\ -1/8 & 0 & 3/8\\ 1/8 & -3/8 & 0 \end{array}\right],$$

and we have (112)

(112) $${\left(W_{X,1}M_{X,1}-W_{X,2}M_{X,2}\right)}^{T}(W_{X,1}M_{X,1}-W_{X,2}M_{X,2})\approx \left[\begin{array}{ccc} 0.0312 & -0.0469 & -0.0469\\ -0.0469 & 0.1406 & 0\\ -0.0469 & 0 & 0.1406 \end{array}\right].$$

The trace of this matrix is (113)

(113) $$\text{Tr}({\left(W_{X,1}M_{X,1}-W_{X,2}M_{X,2}\right)}^{T}(W_{X,1}M_{X,1}-W_{X,2}M_{X,2}))\approx 0.3125.$$

Finally, we get the weighted Frobenius' distance value (114)

(114) $$d_{F,w}(M_{X,1},M_{X,2})=\sqrt{0.3125}\approx 0.5590.$$

If we need to obtain the Frobenius' distance with normalized weights we apply the Equation (97) to get (115):

(115) $$d_{F,\overset{˜}{w}}(M_{X,1},M_{X,2})=\frac{\sqrt{0.3125}}{1-2^{-3}}\approx \frac{\sqrt{0.3125}}{0.8750}\approx 0.6389.$$

**Case 2**: Using reference set $Y=\{y_{1}=B,y_{2}=C,y_{3}=A\}$

Suppose that we work with the same preference orderings ${\text{Pref}}_{1}≜A≻B≻C$ and ${\text{Pref}}_{2}≜A≻C≻B$ with the reference set $Y=\{y_{1}=B,y_{2}=C,y_{3}=A\}$. We get the following ordering matrices (i.e. PSM) $M_{Y,1}$ and $M_{Y,2}$ with their scores $s_{Y,1}$ and $s_{Y,2}$: (116) and (117) respectively

(116) $$M_{Y,1}=\left[\begin{array}{ccc} 0 & 1 & -1\\ -1 & 0 & -1\\ 1 & 1 & 0 \end{array}\right],\;\text{and}\;s_{Y,1}=\left[\begin{array}{c} 0\\ -2\\ 2 \end{array}\right],$$

(117) $$M_{Y,2}=\left[\begin{array}{ccc} 0 & -1 & -1\\ 1 & 0 & -1\\ 1 & 1 & 0 \end{array}\right],\;\text{and}\;s_{Y,2}=\left[\begin{array}{c} -2\\ 0\\ 2 \end{array}\right].$$

Sorting elements of score vectors $s_{Y,1}$ and $s_{Y,2}$ by descending order yields (118)

(118) $$s_{Y,1}^{sorted}=\left[\begin{array}{c} 2\\ 0\\ -2 \end{array}\right],\;\text{and}\;s_{Y,2}^{sorted}=\left[\begin{array}{c} 2\\ 0\\ -2 \end{array}\right].$$

The unitary permutation matrices $V_{Y,1}$ and $V_{Y,2}$ are respectively given by (119) and (120)

(119) $$V_{Y,1}=\left[\begin{array}{ccc} 0 & 1 & 0\\ 0 & 0 & 1\\ 1 & 0 & 0 \end{array}\right],$$

and

(120) $$V_{Y,2}=\left[\begin{array}{ccc} 0 & 0 & 1\\ 0 & 1 & 0\\ 1 & 0 & 0 \end{array}\right].$$

The weighting vectors $w_{Y,1}$ and $w_{Y,2}$ are given by formulas (92) - (93) (with replacing *X* by *Y* in notations), and we get using the same importance weights vector $w={\left[w_{1}=1/2,w_{2}=1/4,w_{3}=1/8\right]}^{T}$ (121) and (122)

(121) $$w_{Y,1}=V_{Y,1}w=\left[\begin{array}{ccc} 0 & 0 & 1\\ 1 & 0 & 0\\ 0 & 1 & 0 \end{array}\right]\left[\begin{array}{c} 1/2\\ 1/4\\ 1/8 \end{array}\right]=\left[\begin{array}{c} 1/4\\ 1/8\\ 1/2 \end{array}\right],$$

(122) $$w_{Y,2}=V_{Y,2}w=\left[\begin{array}{ccc} 0 & 1 & 0\\ 0 & 0 & 1\\ 1 & 0 & 0 \end{array}\right]\left[\begin{array}{c} 1/2\\ 1/4\\ 1/8 \end{array}\right]=\left[\begin{array}{c} 1/8\\ 1/4\\ 1/2 \end{array}\right].$$

Based on $w_{Y,1}$ and $w_{Y,2}$ one gets the weighting matrices (123) and (124)

(123) $$W_{Y,1}=diag(w_{Y,1})=\left[\begin{array}{ccc} w_{2} & 0 & 0\\ 0 & w_{3} & 0\\ 0 & 0 & w_{1} \end{array}\right]=\left[\begin{array}{ccc} 1/4 & 0 & 0\\ 0 & 1/8 & 0\\ 0 & 0 & 1/2 \end{array}\right],$$

(124) $$W_{Y,2}=diag(w_{Y,2})=\left[\begin{array}{ccc} w_{3} & 0 & 0\\ 0 & w_{2} & 0\\ 0 & 0 & w_{1} \end{array}\right]=\left[\begin{array}{ccc} 1/8 & 0 & 0\\ 0 & 1/4 & 0\\ 0 & 0 & 1/2 \end{array}\right].$$

The matrix products $W_{Y,1}M_{Y,1}$ (125) and $W_{Y,2}M_{Y,2}$ (126)

(125) $$W_{Y,1}M_{Y,1}=\left[\begin{array}{ccc} 0 & 1/4 & -1/4\\ -1/8 & 0 & -1/8\\ 1/2 & 1/2 & 0 \end{array}\right],$$

and

(126) $$W_{Y,2}M_{Y,2}=\left[\begin{array}{ccc} 0 & -1/8 & -1/8\\ 1/4 & 0 & -1/4\\ 1/2 & 1/2 & 0 \end{array}\right].$$

Their difference is (127)

(127) $$W_{Y,1}M_{Y,1}-W_{Y,2}M_{Y,2}=\left[\begin{array}{ccc} 0 & 3/8 & -1/8\\ -3/8 & 0 & 1/8\\ 0 & 0 & 0 \end{array}\right],$$

and we have (128)

(128) $${\left(W_{Y,1}M_{Y,1}-W_{Y,2}M_{Y,2}\right)}^{T}(W_{Y,1}M_{Y,1}-W_{Y,2}M_{Y,2})\approx \left[\begin{array}{ccc} 0.1406 & 0 & -0.0469\\ 0 & 0.1406 & -0.0469\\ -0.0469 & -0.0469 & 0.0312 \end{array}\right].$$

The trace of this matrix is (129)

(129) $$\text{Tr}({\left(W_{Y,1}M_{Y,1}-W_{Y,2}M_{Y,2}\right)}^{T}(W_{Y,1}M_{Y,1}-W_{Y,2}M_{Y,2}))\approx 0.3125.$$

Finally we get (130)

(130) $$d_{F,w}(M_{Y,1},M_{Y,2})=\sqrt{0.3125}\approx 0.5590.$$

Or if we need to have distance with normalized weights (131):

(131) $$d_{F,\overset{˜}{w}}(M_{Y,1},M_{Y,2})=\frac{\sqrt{0.3125}}{1-2^{-3}}\approx \frac{\sqrt{0.3125}}{0.8750}\approx 0.6389.$$

We have $d_{F,w}(M_{Y,1},M_{Y,2})=d_{F,w}(M_{X,1},M_{X,2})$, which shows that the IUIP indeed works with this weighted Frobenius' distance as expected (see Theorem). Moreover we can check that $M_{Y,i}=U^{-1}M_{X,i}U$ and $W_{Y,i}=U^{-1}W_{X,i}U$ for i=1,2 using the unitary matrix ***U*** characterizing the permutation from the reference set *X* to the reference set *Y*, which is given in this example by (132)

(132) $$U=\left[\begin{array}{ccc} 0 & 0 & 1\\ 1 & 0 & 0\\ 0 & 1 & 0 \end{array}\right].$$

### 5.8 Example 4: $d_{F,w}$ between tied rankings

We briefly show an example where ties occur in the preference orderings. We still consider 3 objects *A*, *B* and *C* with preference orderings ${\text{Pref}}_{1}≜(A=C)≻B$ and ${\text{Pref}}_{2}≜C≻(A=B)$. We work with the reference set $X=\{x_{1}=A,x_{2}=B,x_{3}=C\}$. Because there are 3 objects, we work a priori with importance weights vector $w={\left[w_{1},w_{2},w_{3}\right]}^{T}$, and as previously we take $w={\left[w_{1}=1/2,w_{2}=1/4,w_{3}=1/8\right]}^{T}$. In this example, we have (133) and (134)

(133) $$M_{X,1}=\left[\begin{array}{ccc} 0 & 1 & 0\\ -1 & 0 & -1\\ 0 & 1 & 0 \end{array}\right],\;\text{and}\;s_{X,1}=\left[\begin{array}{c} 1\\ -2\\ 1 \end{array}\right],$$

(134) $$M_{X,2}=\left[\begin{array}{ccc} 0 & 1 & 1\\ -1 & 0 & 0\\ -1 & 0 & 0 \end{array}\right],\;\text{and}\;s_{X,2}=\left[\begin{array}{c} 2\\ -1\\ -1 \end{array}\right].$$

Sorting elements of score vectors $s_{X,1}$ and $s_{X,2}$ by descending order yields (135)

(135) $$s_{X,1}^{sorted}=\left[\begin{array}{c} 1\\ 1\\ -2 \end{array}\right],\;\text{and}\;s_{X,2}^{sorted}=\left[\begin{array}{c} 2\\ -1\\ -1 \end{array}\right].$$

Because of ties, the previous method cannot be directly applied, and some additional manipulations have to be done to make correctly the weighting assignment of score values when some score values are equal. For this, we must adapt the values of the importance weights vector ***w*** to take into account the multiplicity of score values in their descending order. This adaptation is needed for each preference ordering where ties occur. More precisely, in our example, one sees that the unitary permutation matrix $V_{X,1}$ such that $s_{X,1}=V_{X,1}s_{X,1}^{sorted}$ is not unique and the two matrices $V_{X,1}^{a}$ or $V_{X,1}^{b}$ can be chosen (136)

(136) $$V_{X,1}^{a}=\left[\begin{array}{ccc} 1 & 0 & 0\\ 0 & 0 & 1\\ 0 & 1 & 0 \end{array}\right],\;\text{and}\;V_{X,1}^{b}=\left[\begin{array}{ccc} 0 & 1 & 0\\ 0 & 0 & 1\\ 1 & 0 & 0 \end{array}\right].$$

Similarly, the unitary permutation $V_{X,2}$ such that $s_{X,2}=V_{X,2}s_{X,2}^{sorted}$ is not unique because of the tie, and the two matrices $V_{X,2}^{a}$ or $V_{X,2}^{b}$ can also be chosen (137)

(137) $$V_{X,2}^{a}=\left[\begin{array}{ccc} 1 & 0 & 0\\ 0 & 1 & 0\\ 0 & 0 & 1 \end{array}\right],\;\text{and}\;V_{X,2}^{b}=\left[\begin{array}{ccc} 1 & 0 & 0\\ 0 & 0 & 1\\ 0 & 1 & 0 \end{array}\right].$$

The adaptation (i.e., modification) of importance vector $w={\left[w_{1},w_{2},w_{3}\right]}^{T}$ is necessary to apply to calculate the weighted Frobenius' distance. This is done using the multiplicity of score values and their ranks in the $s_{X,1}^{sorted}$ and $s_{X,2}^{sorted}$ vectors. More precisely, because the multiplicity of the first best score (its value is 1) in $s_{X,1}^{sorted}$ the vector $w={\left[w_{1},w_{2},w_{3}\right]}^{T}$ must be replaced by $w^{\prime }={\left[w_{1},w_{1},w_{2}\right]}^{T}$. Also because of the multiplicity of the second best score (its value is -1) in $s_{X,2}^{sorted}$ the vector $w={\left[w_{1},w_{2},w_{3}\right]}^{T}$ must be replaced by $w^{\prime \prime }={\left[w_{1},w_{2},w_{2}\right]}^{T}$. With these adaptations, we get (138) and (139)

(138) $$w_{X,1}=V_{X,1}^{a}w^{\prime }=V_{X,1}^{b}w^{\prime }=\left[\begin{array}{c} 1/2\\ 1/4\\ 1/2 \end{array}\right],$$

(139) $$w_{X,2}=V_{X,2}^{a}w^{\prime \prime }=V_{X,2}^{b}w^{\prime \prime }=\left[\begin{array}{c} 1/2\\ 1/4\\ 1/4 \end{array}\right]$$

Based on $w_{X,1}$ and $w_{X,2}$ one gets the weighting matrices (140) and (141) respectively

(140) $$W_{X,1}=diag(w_{X,1})=\left[\begin{array}{ccc} w_{1} & 0 & 0\\ 0 & w_{2} & 0\\ 0 & 0 & w_{1} \end{array}\right]=\left[\begin{array}{ccc} 1/2 & 0 & 0\\ 0 & 1/4 & 0\\ 0 & 0 & 1/2 \end{array}\right],$$

(141) $$W_{X,2}=diag(w_{X,2})=\left[\begin{array}{ccc} w_{1} & 0 & 0\\ 0 & w_{2} & 0\\ 0 & 0 & w_{2} \end{array}\right]=\left[\begin{array}{ccc} 1/2 & 0 & 0\\ 0 & 1/4 & 0\\ 0 & 0 & 1/4 \end{array}\right].$$

The matrix products $W_{X,1}M_{X,1}$ and $W_{X,2}M_{X,2}$ are (142)

(142) $$W_{X,1}M_{X,1}=\left[\begin{array}{ccc} 0 & 1/2 & 0\\ -1/4 & 0 & -1/4\\ 0 & 1/2 & 0 \end{array}\right],$$

and (143)

(143) $$W_{X,2}M_{X,2}=\left[\begin{array}{ccc} 0 & 1/2 & 1/2\\ -1/4 & 0 & 0\\ -1/4 & 0 & 0 \end{array}\right].$$

Their difference is (144)

(144) $$W_{X,1}M_{X,1}-W_{X,2}M_{X,2}=\left[\begin{array}{ccc} 0 & 0 & -1/2\\ 0 & 0 & -1/4\\ 1/4 & 1/2 & 0 \end{array}\right],$$

and we have (145)

(145) $${\left(W_{X,1}M_{X,1}-W_{X,2}M_{X,2}\right)}^{T}(W_{X,1}M_{X,1}-W_{X,2}M_{X,2})\approx \left[\begin{array}{ccc} 1/16 & 1/8 & 0\\ 1/8 & 1/4 & 0\\ 0 & 0 & \left(1/4)+(1/16\right) \end{array}\right].$$

The trace of this matrix is (146)

(146) $$\text{Tr}({\left(W_{X,1}M_{X,1}-W_{X,2}M_{X,2}\right)}^{T}(W_{X,1}M_{X,1}-W_{X,2}M_{X,2}))=(1/16)+(1/4)+(1/4)+(1/16)=(1/8)+(1/2)=0.625.$$

Finally, we get the weighted Frobenius' distance value (147)

(147) $$d_{F,w}(M_{X,1},M_{X,2})=\sqrt{0.625}\approx 0.7906,$$

and if it is required to have a distance with normalized weights, we can calculate it as follows (148):

(148) $$d_{F,\overset{˜}{w}}(M_{X,1},M_{X,2})=\frac{d_{F,w}(M_{X,1},M_{X,2})}{1-2^{-3}}=\frac{\sqrt{0.625}}{0.8750}=0.9035$$

Therefore, we see that weighted Frobenius' distance can be calculated even if the preference orderings include ties, but the calculation is a bit more complicated than with strict preference orderings because of the necessity of adapting the importance weights vectors for taking into account the multiplicity of score values in the score vectors (if any). This adaptation can, however, be done automatically in the correct programming code of this method.

Note It is worth noting that $d_{F,w}$ provides the same result as $d_{F}$ when all the components of importance weights vector ***w*** are equal to one. This can be easily verified in our examples 3 and 4, taking $w={\left[1,1,1\right]}^{T}$ and comparing with the result that we obtain using Frobenius' distance formula (47). For example 3 we get $d_{F}=d_{F,w={\left[1,1,1\right]}^{T}}=2.8284$, and for example 4 we get $d_{F}=d_{F,w={\left[1,1,1\right]}^{T}}=2$. It is also worth noting that when working with the normalized distances, the normalization of weights has no impact on the result because one always has (149):

(149) $${\overset{˜}{d}}_{F,\overset{˜}{w}}=\frac{d_{F,\overset{˜}{w}}}{d_{F,\overset{˜}{w}}^{\max}}=\frac{d_{F,w}/(\sum_{i=1}^{n}w(i))}{d_{F,w}^{\max}/(\sum_{i=1}^{n}w(i))}=\frac{d_{F,w}}{d_{F,w}^{\max}}={\overset{˜}{d}}_{F,w}.$$

## 6 Conclusion and perspectives

In this paper, we proposed a new effective distance between rankings based on the Frobenius' norm of the square matrix, which satisfies the invariance under the indexing principle, i.e., it returns the same results with no regard to the order of labels in evaluated sets. The approach is mainly intended to use rankings represented as indexes of the ordered set, which is a more natural way for most people. However, it can also be used with rankings represented by values and provides stable results. Moreover, the proposed approach can deal with ties and can be extended to calculate the weighted distance between two rankings. We have also shown the difference between Frobenius' distance and Kemeny's distance, although they are based on the same definition of ordering matrices.

In future works, we plan to examine how this approach performs in real-life decision-making problems and compare the Frobenius' distance with Kemeny's distance and correlation coefficients used in the literature. Because of the useful properties of the Frobenius' distance, it could be potentially used in distance-based machine-learning algorithms, such as clustering or classification, therefore it would be interesting to investigate such applications too. Another interesting direction of future research is preparing the simulation to check how specific changes in the ranking will influence distance. Finally, it would be very interesting to see if it is possible to extend this approach to uncertain and incomplete rankings.

## CRediT authorship contribution statement

**Jean Dezert:** Writing – original draft, Visualization, Validation, Supervision, Software, Methodology, Investigation, Formal analysis, Conceptualization. **Andrii Shekhovtsov:** Writing – review & editing, Writing – original draft, Visualization, Validation, Software, Methodology, Investigation, Formal analysis, Conceptualization. **Wojciech Sałabun:** Writing – review & editing, Writing – original draft, Validation, Supervision, Project administration, Methodology, Investigation, Funding acquisition, Formal analysis, Conceptualization.

## Declaration of Competing Interest

The authors declare that they have no known competing financial interests or personal relationships that could have appeared to influence the work reported in this paper.

## Footnotes

8 The double subscript F,X introduced in $d_{F,X}^{\max}$ is necessary to indicate explicitly the reference set we are working with, i.e., the set *X* here.

## Footnotes

1 The symbol ≜ means *equals by definition*.

2 Where referring to a set we implicitly refer to a non-ordered set of the naive set theory [15], otherwise we will specify *ordered set* whenever necessary.

3 We omit here the index *s* of the expert for the simplicity of notation because we consider only one expert in this example.

4 Kemeny's ordering matrix coincides with the PSM whose elements are defined by (25).

5 We use the subscript K in our notation to refer to Kemeny.

6 We use the subscript F in our notation to refer to Frobenius.

7 See the definition of similar matrices in [23].

9 For simplicity and for limiting the number of cases to browse, we do not consider all possible ties that can occur.

10 Verify that $d_{F,w}(M_{Y,1},M_{Y,2})=d_{F,w}(M_{X,1},M_{X,2})$ by taking into account that $M_{Y,i}=U^{-1}M_{X,i}U$ and $W_{Y,i}=U^{-1}W_{X,i}U$ for i=1,2 and that ***U*** is a unitary permutation matrix such that $U^{T}U=I_{n\times n}$.

11 In this example $s_{X,1}^{sorted}=s_{X,1}$ because the elements of $s_{X,1}$ are already in (vertical) descending order.
